# Efficient and real-time perception: a survey on end-to-end event-based object detection in autonomous driving

**DOI:** 10.3389/frobt.2025.1674421

**Published:** 2025-11-03

**Authors:** Kamilya Smagulova, Ahmed Elsheikh, Diego A. Silva, Mohammed E. Fouda, Ahmed M. Eltawil

**Affiliations:** 1 Communication and Computing Systems Lab, Computer, Electrical and Mathematical Sciences and Engineering Division, King Abdullah University of Science and Technology, Thuwal, Saudi Arabia; 2 Mathematics and Engineering Physics Department, Faculty of Engineering, Cairo University, Giza, Egypt; 3 Compumacy for Artificial Intelligence Solutions, Cairo, Egypt

**Keywords:** event-based camera, neuromorphic camera, autonomous driving, object detection, event-based dataset, benchmarking

## Abstract

Autonomous driving has the potential to enhance driving comfort and accessibility, reduce accidents, and improve road safety, with vision sensors playing a key role in enabling vehicle autonomy. Among existing sensors, event-based cameras offer advantages such as a high dynamic range, low power consumption, and enhanced motion detection capabilities compared to traditional frame-based cameras. However, their sparse and asynchronous data present unique processing challenges that require specialized algorithms and hardware. While some models originally developed for frame-based inputs have been adapted to handle event data, they often fail to fully exploit the distinct properties of this novel data format, primarily due to its fundamental structural differences. As a result, new algorithms, including neuromorphic, have been developed specifically for event data. Many of these models are still in the early stages and often lack the maturity and accuracy of traditional approaches. This survey paper focuses on end-to-end event-based object detection for autonomous driving, covering key aspects such as sensing and processing hardware designs, datasets, and algorithms, including dense, spiking, and graph-based neural networks, along with relevant encoding and pre-processing techniques. In addition, this work highlights the shortcomings in the evaluation practices to ensure fair and meaningful comparisons across different event data processing approaches and hardware platforms. Within the scope of this survey, system-level throughput was evaluated from raw event data to model output on an RTX 4090 24GB GPU for several state-of-the-art models using the GEN1 and 1MP datasets. The study also includes a discussion and outlines potential directions for future research.

## 1 Introduction

Autonomous vehicles, powered by Autonomous Driving (AD) technologies, are rapidly expanding their presence in the market. Autonomy in the context of AD systems refers to a vehicle’s capability to independently execute critical driving tasks, including object detection, path planning, motion prediction, and vehicle control functions such as steering, braking, and acceleration. This progress is largely enabled by breakthroughs in artificial intelligence (AI), machine learning, computer vision, robotics, and sensor technology. The effective operation of Autonomous Driving Systems (ADS) relies on key functions such as perception, decision-making, and control. The perception system allows the vehicle to sense and interpret its environment in real time, enabling timely and appropriate responses (Messikommer et al., 2022). It collects data from a variety of sensors, including cameras, LiDARs, and radars, to acquire and understand the surrounding environment. The raw sensor data are then processed to perform critical tasks such as object detection, segmentation, and classification, providing essential information for high-level decision making in various applications, including self-driving cars, drones, robotics, wireless communication, and augmented reality (El Madawi et al., 2019; Petrunin and Tang, 2023; Fabiani et al., 2024; Wang Y. et al., 2025). The major players in the field of ADS are Waymo, Tesla, Uber, BMW, Audi, Apple, Lyft Baidu and others (Johari and Swami, 2020; Kosuru and Venkitaraman, 2023; Zade et al., 2024). In particular, Waymo offers “robotaxi” services in major US cities, including Phoenix, Arizona, San Francisco, California. It relies on the fusion of cameras, radar, and LiDAR to navigate in urban surroundings. Tesla implemented its Autopilot system, which functions similarly to an airplane’s autopilot, assisting with driving tasks while the driver remains responsible for full control of the vehicle. Its system eliminates LiDAR and functions based on advanced camera and AI technologies. BMW, in its BMWi Vision Dee system, is working toward integrating augmented reality and human-machine interaction (Suarez, 2025).

Among sensors used in the AD perception system, LiDAR offers high accuracy but suffers from high latency. Radar, on the other hand, provides low latency but lacks precision (Wang H. et al., 2025). Traditional frame-based cameras, which are currently the dominant type (Liu et al., 2024), face challenges in dynamic environments where lighting conditions change rapidly or where extremely high-speed motion is involved. The typical dynamic range of frame-based cameras is around 60 dB (Gallego et al., 2020), and in the high-quality frame cameras, it does not exceed 95 dB (Chakravarthi et al., 2025). The power consumption of these cameras is 1–2 W with a data rate around 30–300 MB/s and a latency of 10–100 ms (Xu et al., 2025). Therefore, recently introduced event-based cameras have gained attention for their distinct operating principles, which are inspired by biological vision systems. This approach emulates the way the brain and nervous system process sensory input, inherently exhibiting neuromorphic properties (Lakshmi et al., 2019). Unlike traditional frame-based cameras that capture the entire scene at fixed intervals, event-based cameras detect changes in brightness at each pixel asynchronously and record events only when a change occurs (Kryjak, 2024; Reda et al., 2024). As a result, they offer faster update rates in the range of 1–10 μ s per event, higher dynamic range exceeding 100 dB, and low power consumption typically around 10–100 mW (Xu et al., 2025). Additionally, eliminating redundant information from static background scenes reduces memory usage with time resolution around 0.1–2 MB/s, depending on the scene (Xu et al., 2025; Chakravarthi et al., 2025). Currently, interest in the event-based domain continues to grow, driving the development of new event-based cameras by hardware vendors, the creation of new datasets and algorithms, and the introduction of simulators specifically designed for the generation and processing of event-driven data (Chakravarthi et al., 2025).

Object detection is a fundamental component of the perception system and plays a vital role in ensuring safe navigation in autonomous driving (Balasubramaniam and Pasricha, 2022). The ability to accurately and promptly identify nearby vehicles, pedestrians, cyclists, and static obstacles is crucial for informed decision-making. Event-based sensors are particularly well-suited for high-speed motion and challenging lighting conditions, offering robustness to motion blur, low latency, and high temporal resolution. This responsiveness enables more precise and timely object recognition, making them a strong candidate for enhancing perception in autonomous vehicles (Zhou and Jiang, 2024). Notably, some of the earliest datasets collected with event-based cameras were captured in driving scenarios, highlighting their relevance for real-world autonomous navigation. These include N-Cars (Sironi et al., 2018), DDD17 (Binas et al., 2017), DDD20 (Hu et al., 2020) datasets. Furthermore, the first large-scale real-world datasets focused on object detection, GEN1 (De Tournemire et al., 2020) and 1MP (Fei-Fei et al., 2004), were specifically designed for this task and are widely accepted as benchmarks for evaluating models.

Despite promising features of event-based cameras, modern processing systems and algorithms are not fully suitable or ready to process sparse spatiotemporal data produced by such sensors. Most traditional computer vision pipelines and Deep Neural Network (DNN) models are designed for frame-based data, where information is structured as sequential images (Perot et al., 2020; Messikommer et al., 2020). In addition, there are significantly fewer event-based datasets available compared to traditional frame-based datasets. Nevertheless, there has been a significant surge in research activity and specialized workshops focused on event-based processing and applications (Chakravarthi et al., 2025; Cazzato and Bono, 2024). This growing interest has also resulted in numerous surveys that review and analyze various aspects of event-based processing and its applications. One of the pioneering surveys in this area was presented in (Lakshmi et al., 2019). It describes the architecture and operating principles of neuromorphic sensors, followed by a brief summary of commercially available event-based cameras, their applications, and relevant algorithms. Due to the limited availability of commercial event-based cameras at the time, the survey includes only early event-based datasets and, for the same reason, explores methods for generating more event data from conventional frame-based sources. A later survey (Gallego et al., 2020) expands the coverage to include both commercially available and prototype event cameras and extends the discussion to include neuromorphic data processors. However, it does not provide information on datasets.

One of the first reviews on event-based neuromorphic vision with a specific focus on autonomous driving is presented in (Chen et al., 2020). The survey discusses the operating principles of event-based cameras, highlighting their advantages and suitability for autonomous driving. It also presents early driving scenario datasets that can be adapted through post-processing for object detection tasks, along with signal processing techniques and algorithms tailored for event-based applications. However, it does not discuss hardware components such as commercially available event-based cameras or neuromorphic processors. The fundamentals of event-based cameras, along with their capabilities, challenges, and the common state-of-the-art cameras, are listed in (Shariff et al., 2024). Most importantly, this survey discusses the appropriate settings for acquiring high-quality data and applications. A more recent survey (Chakravarthi et al., 2025) provided a general overview of research and publication trends in the field, highlighting significant milestones in event-based vision and presenting real-world datasets for various applications and existing cameras. But it lacks information about state-of-the-art preprocessing and processing algorithms and neuromorphic hardware.

Another recent survey on event-based autonomous driving reviewed both early and state-of-the-art publicly available object detection datasets, along with the processing methodologies, classifying them into four main categories, such as traditional Deep Neural Networks (DNNs), bio-inspired Spiking Neural Networks (SNNs), spatio-temporal Graph Neural Networks (GNNs), and multi-modal fusion models (Zhou and Jiang, 2024). There is also a recent survey on event-based pedestrian detection (EB-PD) that evaluates various algorithms using the 1MP and self-collected datasets for the pedestrian detection task, which can be seen as a specific use case of object detection in autonomous driving (Wang H. et al., 2024). A comprehensive and well-structured study on event-based object detection using SNNs, including applications in autonomous driving, can be found in (Iaboni and Abichandani, 2024). It provides an overview of state-of-the-art event-based datasets, as well as SNN architectures and their algorithmic and hardware implementations for object detection. The work also highlights the evaluation metrics that can be used to assess the practicality of SNNs.

Biologically inspired approaches to processing the output of event-based cameras show great promise for their potential to enable energy-efficient and high-speed computing, though they have yet to surpass traditional methods (Shawkat et al., 2024; Iaboni and Abichandani, 2024; Chakravarthi et al., 2025). The study (Shawkat et al., 2024) reviewed approaches involving neuromorphic sensors and processors and pointed out that a major challenge in building fully neuromorphic systems, especially on a single chip, is the lack of solutions for integrating event vision sensors with processors. Similarly, challenges exist in interfacing event-based cameras with systems accelerated using Field Programmable Gate Arrays (FPGAs) or System-on-Chip FPGAs (SoC FPGAs). Additionally, there is limited availability of publicly accessible code, particularly in Hardware Description Languages (HDLs) (Kryjak, 2024).

While effective algorithms and efficient hardware acceleration are crucial for processing event-based data, there are also techniques specifically aimed at enhancing the quality of the event data itself. These methods improve data representation and reduce noise to enhance performance (Shariff et al., 2024). A recent comprehensive survey on deep learning approaches for event-based vision and benchmarking provides a detailed taxonomy of the latest studies, including event quality enhancement and encoding techniques (Zheng et al., 2023). Another survey provides an overview of hardware and software acceleration strategies, with a focus on mobile sensing and a range of application domains (Wang H. et al., 2025). A recent work also surveyed algorithms, hardware, and applications in the event-based domain, highlighting the research gap (Cimarelli et al., 2025).

All aforementioned surveys provide important insights into event-based vision and are summarized in Table 1. Building on these contributions, our survey provides an end-to-end review of event-based vision, covering event-based sensor architectures, key datasets with a focus on object detection in autonomous driving, and the full pipeline from data preprocessing and processing to postprocessing. In addition, we discuss benchmarking metrics designed to support fair and consistent evaluation across different processing approaches and hardware accelerators, aiming to ensure a balanced comparison. This work provides a summary of popular evaluation metrics for object detection models and evaluation of system-level throughput that includes conversion events to the required data format.

**TABLE 1:** Summary of existing surveys on event-based vision: from sensors and algorithms to processors ( ✓ -yes, ✗ - no, ⋆- only autonomous driving).

Paper title	Year	Event-based sensor operation	Available cameras	Event-based datasets	Events preprocessing	Simulators	Neuromorphic processors	Models	Applications
Neuromorphic vision: From sensors to event-based algorithms (Lakshmi et al., 2019)	2018	✓	✓	✓	✓	✓	✗	✗	✓
Event-based Vision: A Survey (Gallego et al., 2020)	2020	✓	✓	✗	✓	✓	✓	✗	✓
Event-Based Neuromorphic Vision for Autonomous Driving: A Paradigm Shift for Bio-Inspired Visual Sensing and Perception (Chen et al., 2020)	2020	✓	✗	✓	✓	✗	✗	✓	★
Event Cameras in Automotive Sensing: A Review (Shariff et al., 2024)	2024	✓	✓	✓	✓	✗	✗	✓	✓
Recent event camera innovations: A survey (Chakravarthi et al., 2025)	2024	✓	✓	✓	✗	✓	✗	✗	✓
Deep Event-based Object Detection in Autonomous Driving: A Survey (Zhou and Jiang, 2024)	2024	✓	✗	✓	✓	✗	✗	✓	★
Research, Applications and Prospects of Event-Based Pedestrian Detection: A Survey (Wang H et al., 2024)	2024	✓	✗	✓	✓	✗	✗	✓	★
Event-based Spiking Neural Networks for Object Detection: A Review of Datasets, Architectures, Learning Rules, and Implementation (Iaboni and Abichandani, 2024)	2024	✗	✗	✓	✓	✗	✗	✓	✓
Review of neuromorphic processing for vision sensor (Shawkat et al., 2024)	2024	✓	✗	✗	✓	✗	✓	✓	✗
Event-based vision on FPGAs – a survey (Kryjak, 2024)	2024	✓	✗	✗	✓	✓	✓	✗	✗
Deep learning for event-based vision: A comprehensive survey and benchmarks (Zheng et al., 2023)	2024	✗	✗	✓	✓	✗	✗	✓	✓
An Application-Driven Survey on Event-Based Neuromorphic Computer Vision (Cazzato and Bono, 2024)	2024	✓	✗	✗	✗	✗	✗	✗	✓
Towards Mobile Sensing with Event Cameras on High-agility Resource-constrained Devices: A Survey (Wang et al., 2025a)	2025	✓	✓	✓	✓	✓	✓	✓	✓
Hardware, Algorithms, and Applications of the Neuromorphic Vision Sensor: a Review	2025	✓	✓	✓	✓	✓	✓	✓	✓
This work	2025	✓	✓	✓	✓	✓	✓	✓	★

The structure of the paper is outlined as follows: Section 2 introduces the fundamental concepts of autonomous driving systems and explains the distinctions between different levels of driving automation. It also highlights the role of object detection in supporting autonomous driving functionality. Section 3 provides a brief overview of the available event-based datasets and their acquisition methods. In particular, Section 3.1 introduces the fundamentals of event-based sensors and highlights notable commercially available models. Section 3.3 explores the characteristics of event-based datasets, covering both early-stage research datasets and real-world as well as synthetic datasets, with an emphasis on autonomous driving scenarios. Section 4 introduces the evaluation metrics and focuses on the neuromorphic processing pipeline, detailing state-of-the-art event-based object detection architectures, their classification, relevant event encoding techniques, and data augmentation methods. Sections 2–4 cover the fundamentals of object detection and event-data acquisition, making the survey accessible to a broader audience, including researchers who are new to event-based object detection. Section 5 presents a system-level evaluation of event-based object detectors and summarizes the performance of models discussed in Section 4.2. Additionally, it addresses missing aspects in end-to-end evaluation. Finally, Section 6 offers a discussion.

## 2 Autonomous driving systems

The Society of Automotive Engineers (SAE) defines six levels of autonomy in autonomous driving systems (Zhao et al., 2025). These levels are based on who performs the Dynamic Driving Task (DDT), either the driver or the system. A key part of DDT is Object and Event Detection and Response (OEDR), which refers to the system’s ability to detect objects in the environment, such as vehicles, pedestrians, and traffic signs, and respond appropriately. Level 0 of the SAE indicates no autonomy and full manual driving, while Levels 1 through 5 represent increasing degrees of automation, with each level incorporating more advanced autonomous features. As the level of autonomy increases, the vehicle’s reliance on intelligent systems becomes more critical for ensuring safe and efficient navigation in complex environments (Zhao et al., 2025; Balasubramaniam and Pasricha, 2022). The SAE also introduced the concept of the Operational Design Domain (ODD), a key characteristic of a driving automation system. Defined by the system’s manufacturer, the ODD outlines the specific conditions, such as geographic area, road type, weather, and traffic scenarios under which the autonomous system is intended to operate ERTRAC (2019). Overall, the SAE levels describe the degree of driver involvement and the extent of autonomy, while the ODD defines the specific conditions where and when that autonomy can be applied (Warg et al., 2023). Table 2 summarizes SAE Levels of automation for on-road vehicles and the role of object detection. Clearly, as the level of autonomy increases, the importance of object detection becomes increasingly critical.

**TABLE 2:** The SAE levels of autonomy and role of object detection.

SAE levels	Name	SAE levels description	DDT		ODD	Role of object detection
			Lateral and longitudinal motion control	OEDR		
Level 0	No automation	The human driver performs all aspects of the driving task at all times	Driver	Driver	no	Optional. Not required by automation, but may be used for assistance
Level 1	Driver assistance	The system assists with either steering or acceleration/deceleration using info about the environment	Driver and System	Driver	limited	Supports object detection for adaptive functions for either steering and braking or accelerating either lateral or longitudinal motion control
Level 2	Partial driving automation	The system performs steering and acceleration/deceleration, but the driver must monitor and intervene if needed	System	Driver	limited	Required for a lane keeping assist (LKA), an adaptive cruise control (ACC) and environmental perception
Level 3	Conditional driving automation	The system performs all DDT within the defined ODD but requests takeover when necessary	System	System	limited	Essential for scene understanding, obstacle avoidance, and fallback planning
Level 4	High driving automation	The system performs all driving tasks and handles fallback in the defined ODD without requiring human input	System	System	limited	Critical for safe operation; must detect and respond to all obstacles and events
Level 5	Full driving automation	The system performs all driving tasks under all conditions without any human involvement	No human driver	System	unlimited	Mandatory and fully integrated; complete situational awareness required

Most commercial vehicles today operate at Level 2, where the system can control steering and speed. This includes Tesla Autopilot, Ford BlueCruise, Mercedes Drive Pilot (Leisenring, 2022). Waymo has advanced into Level 4, offering fully autonomous services within geofenced urban areas like Phoenix and San Francisco, without a safety driver onboard Ahn (2020). Uber, while investing heavily in autonomy, currently operates at Level 2–3 through partnerships and focuses on integrating automation with human-supervised fleets Vedaraj et al. (2023). Level 5, representing universal, human-free autonomy in all environments, remains a long-term goal for the industry and has not yet been achieved by any company.

The SAE proposes an engineering-centric classification, while there is also a user-centric perspective for vehicle automation classification. According to Koopman, there are four operational modes, which include driver assistance, supervised automation, autonomous operation, and vehicle testing. The latter distinct category is for testing purposes, where the human operator is expected to respond more effectively to automation failures than a typical driver. Mobileye also suggests four dimensions, such as hands-on/hands-off (for steering wheel), eyes-on/eyes-off (the road), driver/no driver, and Minimum Risk Maneuver (MRM) requirement Warg et al. (2023). All of the above-mentioned automation level definitions are focused on driving tasks on-road traffic. There are other dimensions for autonomy classification focused on interaction in various environments, which are not covered in this work.

## 3 Neuromorphic data acquisition and datasets

### 3.1 Event-based sensors

**FIGURE 1:**
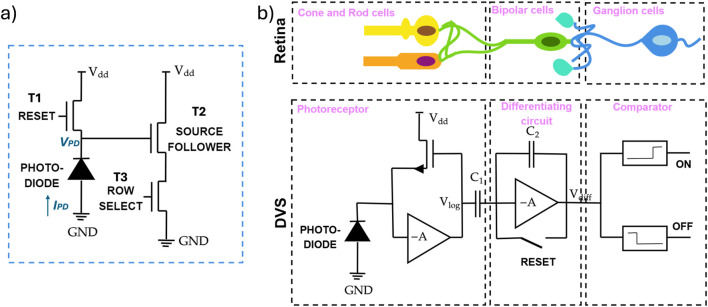
**(a)** Active Pixel Sensor; **(b)** Retina and Dynamic Vision Sensor (DVS).

Traditional image- and video-acquiring technology primarily revolves around frame-based cameras capable of capturing a continuous stream of still pictures at a specific rate. Each still frame consists of a grid of 2D pixels with global synchronization, generated using sensor technologies like Charge-Coupled Devices (CCDs) or Complementary Metal Oxide-Semiconductor (CMOS) sensors. Due to their superior imaging quality, CCDs are favored in specialized fields such as astronomy (Polatoğlu and Özkesen, 2022), microscopy (Faruqi and Subramaniam, 2000), and others. These sensors feature arrays of photodiodes, capacitors, and charge readout circuits that convert incoming light into electrical signals. In contrast, CMOS sensors dominate consumer electronics due to their lower cost and sufficient image quality. CMOS sensors can be designed as either Active Pixel Sensors (APS) or, less commonly, Passive Pixel Sensors (PPS) (Udoy et al., 2024). A basic APS pixel sensor is comprised of a 3-transistor (3-T) cell, which includes a reset transistor $T_{1}$ , a source follower transistor $T_{2}$ , and a row select transistor $T_{3}$ (Figure 1a). In this setup, a reverse-bias photodiode (PD) is used to detect incoming light. During the reset phase, a transistor $T_{1}$ turns on and $V_{PD}$ charges to a reference voltage $V_{\text{DD}}$ . After resetting, $T_{1}$ is turned off and the integration phase begins. During this phase, incident light generates a photocurrent $I_{PD}$ , which gradually discharges voltage $V_{PD}$ . This voltage drop is buffered by source follower $T_{2}$ and, when the row select transistor $T_{3}$ is activated, read by the readout circuit.

However, these technologies generate large amounts of spatiotemporal data, requiring hardware with high processing capabilities and increased power consumption. This has also led to the development of sensors inspired by biological vision (Shawkat et al., 2024). Particularly, a new imaging paradigm inspired by the function of the human retina, located at the back of the eye, has started gaining attention. The sensing in the retina is done by cones and rods of a photoreceptor, which convert light to electrical signals and pass them to ON/OFF bipolar cells and eventually to ganglion cells. The latter two respond to various visual stimuli, such as intensity increments or decrements, colour, or motion. Similar to the retina, pixels in novel event-based cameras generate output independently from each other and only when some changes in the captured scene occur.

There are several approaches to implementing event-based sensors. The first one is the Dynamic Vision Sensor (DVS). Its pixel architecture shown in Figure 1b mimics a biological retina and is comprised of three blocks, such as a photoreceptor, switched capacitor differentiator, and comparator blocks, which act as photoreceptor, bipolar, and ganglion cells. To produce ON and OFF events, DVS measures light intensity change and slope. In particular, at the initial stage, the DVS pixel starts with a reference voltage that corresponds to the logarithmic intensity of previously observed light. When light hits a photodiode, the generated current $I_{PD}$ starts to discharge the voltage $V_{PD}$ . The rate at which the photodiode voltage changes depends on the intensity of the incoming light. The differentiating circuit produces a voltage proportional to the input’s rate of change. Slow changes result in small outputs, while rapid changes cause voltage spikes. The comparator circuit evaluates the differentiated signal against a fixed threshold and outputs a HIGH or LOW signal based on the result. The output format of event-based cameras is a stream of tuples $e_{i}$ = ( $t_{i}$ , $x_{i}$ , $y_{i}$ , $p_{i}$ ), which provide information about the time $t_{i}$ when the $i^{th}$ event $e_{i}$ happened, its coordinates $\left(x_{i},y_{i}\right)$ , and polarity $p_{i}$ .

**FIGURE 2:**
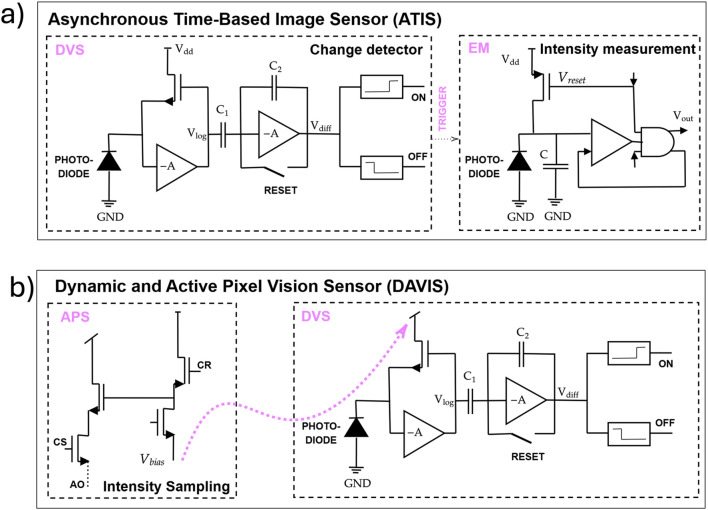
**(a)** Asynchronous Time-Based Image Sensor (ATIS); **(b)** Dynamic and Active Pixel Vision Sensor (DAVIS).

In addition, there are hybrid types of event-based sensors, which include Asynchronous Time Based Image Sensor (ATIS) and DAVIS, shown in Figures 2a,b, respectively. ATIS is a combination of DVS and Time to First Spike (TFS) technologies (Posch et al., 2010). Here, the DVS detects changes in the event stream, while Pulse Width Modulation (PWM) in the Exposure Measurement (EM) component enables the capture of absolute brightness levels. The second photodiode in the ATIS architecture allows it to measure both event intensity and temporal contrast. As a result, ATIS has a larger pixel area compared to DVS and produces enriched tripled data output. The output event of ATIS is $e_{v}$ = $\left(x,y,t,p,e_{\mathrm{lum}},e_{cb},e_{cr}\right)$ , where x,y represent the pixel position, t is the timestamp and p is the event polarity, while $e_{\mathrm{lum}},e_{cb},e_{cr}$ correspond to the YCbCr color components, providing richer scene information (Shawkat et al., 2024; Lesage et al., 2023).

DAVIS is an image sensor comprised of synchronous APS and asynchronous DVS that share a common photodiode, as shown in Figure 2b. It provides multimodal output, which requires data fusion and more complex processing. In particular, a frame-based sampling of the intensities by APS allows for receiving static scene information at regular intervals but leads to higher latency (Shawkat et al., 2024), while DVS produces events in real-time based on changes.

Event-based cameras are typically equipped with control interfaces known as “biases”. These biases configure key components such as amplifiers, comparators, and photodiode circuits, directly impacting latency and event rate. The event bias settings can be adjusted to adapt to specific environmental conditions and to filter out noise (Shariff et al., 2024).

The most recent summary on the commercially available event-based cameras and their specifications can be found in (Gallego et al., 2020; Chakravarthi et al., 2025). The main vendors include iniVation (e.g., DVS128, DVS240, DVS346), Prophesee (e.g., ATIS, Gen3 CD, Gen 3 ATIS, Gen 4 CD, EVK4 HD), CelePixel (e.g., Cele-IV, Cele-V), Samsung (e.g., DVS Gen 2, DVS Gen 3, DVS Gen 4), and Insightness. In addition, (Chakravarthi et al., 2025), provides a list of open-source event-based camera simulators. The notable ones include DAVIS (Mueggler et al., 2017) and Prophesee Video to Event Simulator (Prophesee, 2025). The key event cameras used for the collection of the real-world large-scale event datasets include Prophesee’s GEN1, GEN4, EVK4, and IniVation DAVIS346, whose specifications can be found in Table 3. An important milestone in the field of event-based sensing is the collaboration of Prophesee and Sony, resulting in a hybrid architecture IMX636. This sensor was integrated into industrial camera IDS Imaging uEye XCP EVS (IDS Imaging Development Systems GmbH, 2025), Prophesee EVK4 and EVK5 Evaluation Kits (Chakravarthi et al., 2025), and others.

**TABLE 3:** Key commercial event cameras [adapted from (Gallego et al., 2020; Chakravarthi et al., 2024; Wang H. et al., 2025)].

Output	Parameter	Prophesee ATIS GEN1	IniVation DAVIS346	Prophesee GEN4	Samsung DVS-Gen4	Prophesee EVK4 HD	Prophesee EVK5 HD	uEye XCP EVS
Event output	Spatial Resolution	304 × 240	320 × 240	1280 × 720	1280 × 960	1280 × 720	1280 × 720	1280 × 720
	Temporal Resolution	–	1 μ s	–	-	100 μ s	100 μ s	1 μ s
	Max Throughput	–	12 MEPS	1066 MEPS	1200 MEPS	–	–	-
	Max Bandwidth	–	–	–	–	1.6 Gbps	1.6 Gbps	-
	Latency	3 μ s	< 1 ms	20–150 μ s	150 μ s	–	800 μ s	-
	Dynamic Range	143 dB	120 dB	> 124 dB	100	> 86 dB	> 110 dB	120 dB
	Contrast Sensitivity	13%	14.3%–22.5%	11%	20%	25%	25%	25%
	Pixel Pitch	30 μ m	18.5 μ m	4.86 μ m	4.95 μ m	4.86 μ m	4.86 μ m	4.86 μ m
	Low Light Cutoff	–	–	–	–	0.08 lux	0.08 lux	0.08 lux
Frame output	Spatial Resolution	n/a	346 × 260	n/a	n/a	n/a	n/a	n/a
	Frame Rate	n/a	Up to 40 FPS	n/a	n/a	n/a	n/a	n/a
	FPN	n/a	4.2%	n/a	n/a	n/a	n/a	n/a
	Dark Signal	n/a	18,000 $e^{-}$ /s	n/a	n/a	n/a	n/a	n/a
	Readout Noise	n/a	55 $e^{-}$	n/a	n/a	n/a	n/a	n/a
	Pixel Pitch	n/a	18.5 μ m	n/a	n/a	n/a	n/a	n/a
Other specifi-cations	Power Consumption	50–175 mW	< 700 mW (140 mA @ 5 VDC (USB))	32–84 mW	130 mW	0.5 W via USB	0.5 W via USB	0.5 W via USB
	Year	2011	2017	2020	2020	2022	2023	2025

MEPS, Million Events Per Second; $e^{-}$ , electron; $e^{-}$ /s, electrons per second; dB, decibel; μ s, microseconds; ms, milliseconds; μ m, micrometers; mW, milliwatts; W, watts; mA, milliamperes; FPS, frames per second; Gbps, Gigabits per second; n/a, not applicable.

### 3.2 Synthetic event-based data generation

Slow progress in the event-based domain was caused by the fact that event sensors are both rare and expensive. Furthermore, producing and labeling real-world data is a resource-intensive and time-consuming process. As an alternative, datasets can be generated synthetically (Aliminati et al., 2024). One of the prominent tools for this purpose is the Car Learning to Act (CARLA) simulator (Dosovitskiy et al., 2017), which provides highly realistic virtual environments for autonomous driving. CARLA supports a variety of sensor outputs, including event cameras, RGB cameras, depth sensors, optical flow, and others, enabling the creation of diverse and realistic synthetic event-based datasets.

The Event Camera Simulator (ESIM) is one of the pioneering works in event simulation Rebecq et al. (2018). Its architecture is tightly integrated with the rendering engine and generates events through adaptive sampling, either from brightness changes or pixel displacements. Vid2E Gehrig et al. (2020) follows the same principle and is considered an extension of ESIM. Unlike ESIM, which relies on image input, Vid2E uses video as input. The data generated by Vid2E was evaluated on object recognition and semantic segmentation tasks.

EventGAN generates synthetic events using a Generative Adversarial Network (GAN) (Zhu et al., 2021). The GAN is trained on a pair of frame data and events from the DAVIS sensor. During training, the network is constrained to mimic information present in the real data. To generate events, EventGAN takes input from a pair of grayscale images from existing image datasets.

V2E toolbox creates events from intensity frames Hu et al. (2021). This enabled the generation of event data under bad lighting and motion blur. This contributed to the development of more robust models. V2E produces a sequence of discrete timestamps, whereas real DVS sensors generate a continuous event stream Zhang et al. (2024). Video to Continuous Events Simulator (V2CE) tried to overcome this issue of V2E. V2CE includes two stages: (1) motion-aware event voxels prediction, and (2) voxels to continuous events sampling. Besides, it takes into account the nonlinear characteristics of the DVS camera. Additionally, this work introduced quantifiable metrics to validate synthetic data Zhang et al. (2024).

DVS-Voltmeter allows the generation of synthetic events from high frame-rate videos. It is the first event simulator that took into account physics-based characteristics of real DVS, which include circuit variability and noise Lin et al. (2022). The generated data was evaluated on semantic segmentation and intensity-image reconstruction tasks, demonstrating strong resemblance to real event data.

The ADV2E framework proposed a fundamentally different approach in event generation Jiang et al. (2024). It focuses on analogue properties of pixel circuitry rather than logical behavior. Synthetic events are generated from APS frames. Particularly, emulating an analog low-pass filter allows generating events based on varying cutoff frequencies.

The Raw2Event framework enables the generation of event data from raw frame cameras, producing outputs that closely resemble those of real event-based sensors Ning et al. (2025). It currently generates events from grayscale images, but could be extended to support color event streams. A low-cost solution deployed on Raspberry Pi could also be built on edge AI hardware, enabling lower latency and practical use at the edge.

A recently proposed PyTorch-based library, Synthetic Events for Neural Processing and Integration (SENPI), converts input frames into realistic event-based tensor data Greene et al. (2025). SENPI also includes dedicated modules for event-driven input/output, data manipulation, filtering, and scalable processing pipelines for both synthetic and real event data.

To sum up, most of these tools are rule-based, designed to convert APS-acquired images into synthetic event streams. The only exception is EventGAN, which is learning-based, but it tends to be less reliable and heavily dependent on the quality and diversity of the training data. Among these simulators, ESIM and DVS-Voltmeter stand out for offering the highest realism. Tools like v2e, v2ce, and ADV2E are the most scalable for large dataset generation, while recently introduced Raw2Event is the simplest, lightest, and fastest option. A novel framework, SENPI, offers controlled simulation of event cameras and extended processing features, including data augmentation and manipulation, and algorithmic development.

### 3.3 Event-based datasets

#### 3.3.1 Early event-based datasets

There is a growing variety of neuromorphic datasets that were generated synthetically or recorded in real-world scenarios and cover a wide spectrum of event-based vision tasks, from small-scale classification to real-world autonomous navigation. Depending on the method of capture, they are primarily divided into two categories: ego-motion and static, also known as fixed. Event-based datasets collected from a static/fixed perspective typically focus on the movement of objects or features in the environment, whereas ego-motion datasets emphasize the movement of the observer or camera relative to the scene (Verma et al., 2024).

Early event-based datasets include DVS-converted datasets N-MNIST (Orchard et al., 2015), MNIST-DVS (Serrano-Gotarredona and Linares-Barranco, 2015), CIFAR 10-DVS (Li et al., 2017), N-Caltech101 (Orchard et al., 2015), and N-ImageNet (Kim et al., 2021) are publicly available datasets converted to event-based representation from frame-based static image datasets MNIST (LeCun et al., 1998), CIFAR 10 (Krizhevsky and Hinton, 2009), Caltech101 (Fei-Fei et al., 2004), and ImageNet (Deng et al., 2009). The conversion of frame-based images to an event stream was achieved either by moving the camera, as in case of N-MNIST and N-Caltech101, or by a repeated closed-loop smooth (RCLS) movement of frame-based images, as in MNIST-DVS, CIFAR 10-DVS(Iaboni and Abichandani, 2024; Li et al., 2017). The latter method produces rich local intensity changes in continuous time (Li et al., 2017). The pioneering DVS-captured dataset is DVS128 Gesture. It was generated by natural motion under three lighting conditions, including natural light, fluorescent light, and LED light (He et al., 2020). All of them serve as important benchmark datasets for developing and testing models in the context of event-based vision. However, only N-Caltech includes bounding box annotations, making it the most suitable dataset for the object detection task, which is the primary focus of this survey.

#### 3.3.2 Event-based datasets with autonomous driving context

There is a variety of DVS-captured datasets, each focusing on different aspects of event-based vision and application domains. Table 4 summarizes commonly used event-based datasets related to autonomous driving. These datasets differ in spatial and temporal resolution, collection sensor types, and environmental conditions such as lighting and weather. In addition to the dataset collection process, dataset labeling also plays an essential role in effective object detection. However, annotating event-based data at every timestamp is highly resource-intensive (Wu et al., 2024). Moreover, event data with low spatial or temporal resolution often results in poor quality and limited utility, while higher-resolution data significantly increases memory requirements. Although high temporal resolution improves the tracking of fast-moving objects, it also introduces greater sensitivity to noise. To balance these trade-offs, different datasets adopted different labeling frequencies.

**TABLE 4:** Event-based datasets with autonomous driving context.

Dataset	Year	Camera	Sensor	Modality	Prespective	Resolution	Classes	# Bounding boxes	Labeling frequency	Duration	Scenarios	Different weather conditions	Different lightning conditions
N-CARS (Sironi et al., 2018)	2017	Prophesee	ATIS	events	ego	n/a	Cars, non-Cars	12.3 K, 11.6 K	n/a	80 min	Driving	n/a	n/a
DDDR17 (Binas et al., 2017)	2017	DAVIS346B	APS + DVS	events	ego	n/a	no	no	no	12 h	Driving	✓	✓
DDDR20 (Hu et al., 2020)	2017	DAVIS	APS + DVS	events	ego	n/a	no	no	no	51 h	Driving	✓	✓
Gen1 (De Tournemire et al., 2020)	2020	Prophesee ATIS GEN1	ATIS	events	ego	304×240	Cars, Pedestrians	228 K, 28 K	1–4 Hz	39 h	Driving	✓	✓
1MP (Perot et al., 2020)	2020	Prophesee 1MP (GEN4) + Go Pro	APS + DVS	events, frames	ego	1280×720	Cars, Pedestrians, Two-wheelers	16.3 M, 8.5 M, 1.1 M	60 Hz	14 h	Driving	✓	✓
PKU-DAVIS-SOD (Li et al., 2023)	2022	DAVIS346	APS + DVS	events, frames, e2vid reconstructions	ego	346 × 260	Cars, Pedestrians, Two-wheelers	1.08 M (total)	25 Hz	n/a	Driving	✗	✓
PEDRo (Boretti et al., 2023)	2023	DAVIS346	APS + DVS	events	ego	304×240	Pedestrians	43 K	25 Hz	0.6 h (220 sequences)	Robotics	✓	✓
eTraM (Verma et al., 2024)	2024	Prophesee EVK4 HD	APS + DVS	events, frames	fixed	1280 × 720	Cars, Pedestrians, Tracks, Buses, Trams, Bicycles, Bikes, Wheelchairs	over 2 M (total)	30 Hz	10 h	static Traffic monitoring	✓	✓
SEVD (Aliminati et al., 2024)	2024	CARLA simulator	Multiple DVS	events	ego, fixed	1280×960	Car, Truck, Van, Bicycle, Motorcycle, Pedestrian	over 9 M total	n/a	58 h (total)	Driving, Traffic monitoring	✓	✓
eCARLA-scenes (Mansour et al., 2024)	2024	CARLA simulator	DVS, grayscale, optical flow	events, frames, motion field	ego	260×346	Pedestrians Vehicles	no	n/a	31 sequences	Driving, Traffic monitoring	✓	✓

The DDD17 (Davis Driving Dataset, 2017; Binas et al., 2017) was among the first datasets specifically created for this purpose and includes 12 h of recording. It was collected from German and Swiss roads at speeds ranging from 0 to 160 km/h using a DAVIS346B prototype camera with a resolution of 346×260 pixels. The camera had APS and DVS sensors, which allowed capturing both event- and frame-based data through the same optics. It consists of a continuous event stream captured under various weather and lighting conditions and was used for steering angle prediction. Since the DDD17 is not categorized into specific object classes, its direct utilization in object detection tasks is infeasible without pre-processing and adaptation. An extended version of DDD17 is DDD20 (Hu et al., 2020). DDD20 has around 51 h of recordings under various weather and lightning conditions.

Another complex dataset recorded in changing environments is N-Cars (Sironi et al., 2018). It was collected using Prophesee’s ATIS camera mounted behind the windshield of a car and consists of 80 min of video. Then, gray-scale measurements from the ATIS sensor were converted into conventional gray-scale images. ATIS’s luminous intensity measures were used to generate ground-truth annotations. The resulting dataset has two classes, comprised of 12,336 car samples and 11,693 non-car samples.

Three additional event-based datasets focusing on human motion were later introduced: the pedestrian detection dataset, the action recognition dataset, and the fall detection dataset. The event streams, recorded both indoors and outdoors, were converted into frames and annotated using the labelImg tool. The resulting DVS-Pedestrian dataset contains 4,670 annotated frames (Miao et al., 2019).

Prophesee’s GEN1 Automotive Detection Dataset (also called GAD (Crafton et al., 2021)) is the first large-scale real-world event-based labeled dataset that includes both cars and pedestrians (De Tournemire et al., 2020) and is recognized as the first major detection benchmark. The dataset was collected by the Prophesee ATIS GEN 1 sensor with a resolution of 304×240 mounted behind the windshield of a car. GEN1 contains more than 39 h of recordings of various scenes in different lighting and weather conditions. To decrease the gap between frame-based and event-based datasets in supervised tasks such as detection and classification, the obtained dataset was manually labeled at a frequency between 1 and 4 frames per second (FPS). GEN1 is widely utilized for developing and benchmarking event-based vision technologies and processing algorithms. Additionally, since it was recorded using the first generation of event-based vision sensors, the GEN1 dataset exhibits lower resolution and a higher level of inherent noise compared to more recent datasets (Perot et al., 2020).

More detailed environmental mapping is achieved in a 1 Megapixel (1MP) automotive detection dataset (Perot et al., 2020) recorded by an event-based vision sensor with high resolution (1280×720) , making it suitable for detailed spatial analysis (Finateu et al., 2020). In addition to the dataset, a fully automated labeling protocol is implemented, the key concept of which is acquiring data simultaneously with the Prophesee GEN4 event-based camera and an RGB GoPro Hero 6 camera positioned side by side as closely as possible. Then, the bounding boxes from the frame camera images are transferred to the event-based camera output. The 1MP dataset contains 14 h of recordings with around 25 M bounding boxes of pedestrians (8.5 M), cars (16.3 M), and two-wheelers (1.1 M) at 60 FPS, facilitating high-temporal-precision tasks.

PKU-DAVIS-SOD is a multimodal object detection dataset with the focus on challenging conditions. It has 1.08 M bounding boxes for 3 classes, such as cars, pedestrians, and two-wheelers (Li et al., 2023). Compared to GEN1 and 1MP datasets, the PKU-DAVIS-SOD dataset offers moderate resolution (346 × 260). The dataset was collected by DAVIS346 installed on the front windshield of the driving car, and, to capture high-speed objects, a camera was also placed at the side of the road. The data obtained are in three modalities, such as RGB frames, event images, and grayscale images reconstructed from events using E2VID (Rebecq et al., 2019), and were manually annotated at a frequency of 25 FPS.

Person Detection in Robotics (PEDRo) is another event-based dataset primarily designed for robotics, but can also be used in autonomous driving contexts for pedestrian detection. DAVIS346 camera with a resolution of 304×240 was hand-carried to capture people walking and on some occasions, standing still, sitting, or running (Boretti et al., 2023). PEDRo, with manually annotated 43 K bounding boxes (25 FPS), can serve as a valuable resource to mitigate the class imbalance present in the GEN1 and 1MP datasets.

eTraM is one of the recent event-based datasets (Verma et al., 2024). It is a static traffic monitoring dataset recorded by a 1280×720 Prophesee EVK4 HD event camera. The dataset contains 10 h of recordings, providing 2 M bounding box annotations of eight classes, including pedestrians, cars, trucks, buses, trams, bikes, bicycles, and wheelchairs that were manually annotated.

#### 3.3.3 Synthetic event-based datasets

CARLA simulator was used to generate the Synthetic Event-based Vision Dataset (SEVD) (Aliminati et al., 2024) for both multi-view (360°) ego-motion and fixed-camera traffic perception scenarios, providing comprehensive information for a range of event-based vision tasks. The synthetic data sequences were recorded using multiple dynamic vision sensors under different weather and lightning conditions and include several object classes such as car, truck, van, bicycle, motorcycle, and pedestrian.

Additionally, the CARLA simulator, along with the recently developed eWiz a Python-based library for event-based data processing and manipulation, was used to generate the eCARLA-scenes synthetic dataset, which includes four preset environments and various weather conditions (Mansour et al., 2024).

#### 3.3.4 Event-based dataset labeling

Event-based datasets remain underrepresented. Additionally, the accuracy of object detection is influenced by dataset labeling and its temporal frequency. If labels are sparse in time, the model may miss critical information, especially in high-speed scenarios. On the other hand, higher labeling frequency can become redundant in low-motion scenes and is often expensive to implement manually. To address the scarcity of well-labeled event-based datasets, the overlap between event-based and frame-based data can be exploited to generate additional labeled event datasets (Messikommer et al., 2022). In (Perot et al., 2020), event-based and frame-based cameras were paired as in the 1MP dataset. Since frame-based and event-based sensors were placed side by side, a distance approximation was applied afterwards, and labels extracted from the frame-based camera were transferred to event-based data. Another option suggests the generation of event-based data from existing video using video-to-event conversion (Gehrig et al., 2020).

Unlike frame-based cameras, event-based sensors inherently capture motion information. Adoption of Unsupervised Domain Adaptation (UDA) to enable the transfer of knowledge from a labeled source (e.g., image $Y_{\mathrm{img}}$ ) domain to an unlabeled target (e.g., event $Y_{\mathrm{event}}$ ) domain (Messikommer et al., 2022) was proposed in (Messikommer et al., 2022). This method does not require paired data from both sensors, making it possible to leverage labeled frame-based datasets to train models for unlabeled event-based data. Moreover, a single photo is sufficient to transfer labels, eliminating the need for high-frame-rate videos.

Labeling event data directly from sensor output, without relying on corresponding frame-based information, faces its own challenges. In particular, labeling event-based data at each timestep is expensive due to its high temporal resolution. To address this challenge, Label-Efficient Event-based Object Detection (LEOD) was proposed (Wu et al., 2024). LEOD involves pre-training a detector on a small set of labeled data, which is then used to generate pseudo-labels for unlabeled samples. This approach supports both weakly supervised and semi-supervised object detection settings. To improve the accuracy of the pseudo-labels, temporal information was used. Specifically, time-flip augmentation was applied, which enabled model predictions on both the original and temporally reversed event streams. LEOD was evaluated on the GEN1 and 1MP datasets, and it can outperform fully supervised models or be utilized together to enhance their performance.

## 4 Event-based object detection

To a great extent, traditional object detectors can be divided into single-stage detectors and two-stage detectors (Bouraya and Belangour, 2021; Carranza-García et al., 2020). The single-stage detector is comprised of several parts, which typically include an input, a backbone for feature extraction, a detection head, and, optionally, neck layers. Its neck layers are located between the backbone and head layers and consist of several top-down and bottom-up paths to extract multi-scale features for detecting objects of various sizes (Bouraya and Belangour, 2021). A detection head takes the outputs of the backbone and neck and transforms extracted features into a final prediction. You Only Look Once (YOLO) (Hussain, 2024) and Single Shot MultiBox Detector (SSD) (Liu et al., 2016) are examples of Single-stage detectors. YOLO divides the image into a grid and predicts bounding boxes for each cell, while SSD uses multiple feature maps at different scales to detect objects of varying sizes. Two-stage detectors include an additional step before the classification stage, known as the regions of interest (RoI) proposal stage (Carranza-García et al., 2020). This extra stage helps to identify potential object locations for better performance. As a result, single-stage detectors predict object classes and bounding boxes in one pass and provide higher speed, whereas two-stage detectors try to ensure accurate prediction and involve more computational cost.

Unlike frame-based data, the binary event stream is characterized by spatial and temporal sparsity. Handling such data requires high-performing algorithms. The structure of existing event-based object detection models is comprised of a backbone architecture followed by an SSD- or YOLO-based head. Detection model backbone architectures can be classified as dense, spiking, or graph-based, and can often be converted between formats to enhance efficiency during training and inference. Depending on the model architecture, event data may be processed in its raw form or require conversion. Once formatted appropriately, models can operate either asynchronously on raw event streams or at a fixed rate using dense frame or graph-based representations.

**FIGURE 3:**
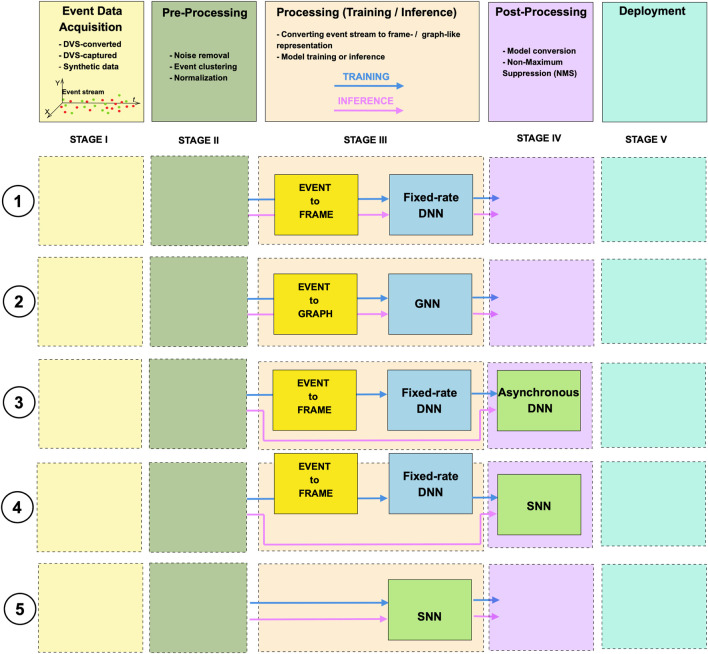
Event-based object detection pipeline: event-data acquisition, pre-processing, processing, post-processing, and deployment. Five types of pipelines based on processing rate and backbone model architecture: fixed-rate dense, fixed-rate graph-based, asynchronous sense, asynchronous spike-based processing dense data, and asynchronous spike-based processing raw events.

Figure 3 summarizes the basic pipeline of event-based object detectors, categorized by the type of model used. While the pipeline can be extended with additional pre- and post-processing stages, in the diagram we focus on the minimal encoding and processing components. The processing stage typically involves converting event data into a specific format, if required, to match the input requirements of the target model and training or inference processes. Based on the type of data processing, these models can be categorized as either event-driven asynchronous (green boxes in Figure 3) or fixed-rate synchronous (blue boxes in Figure 3). Furthermore, based on the backbone model architecture, the networks can be categorized as dense, spiking, or graph-based, resulting in five possible processing pathways within the pipeline. More details on models are provided below in Section 4.2. Although detection models differ in their architectures and processing strategies, it should be noted that they share several common evaluation metrics, with some variations depending on the specific processing approach. In the following sections, we begin by outlining these key evaluation metrics, then introduce state-of-the-art models. We also review existing data augmentation techniques and highlight relevant neuromorphic accelerators.

### 4.1 Evaluation metrics

Evaluation methods applied to event-based object detectors are inherited from frame-based frameworks. The widely adopted one is the COCO (Common Object in Context) metric protocol, which utilizes various performance metrics such as Average Precision (AP), ${\text{AP}}_{50}$ , Average Precision Small (APS), Average Precision Medium (APM), and Average Precision Large (APL) (Perot et al., 2020; Tian et al., 2024). But the key metrics in the evaluation of object detectors include mean Average Precision (mAP) for measuring the accuracy of the object detection, and runtime for measuring the amount of time required to process input.

**FIGURE 4:**
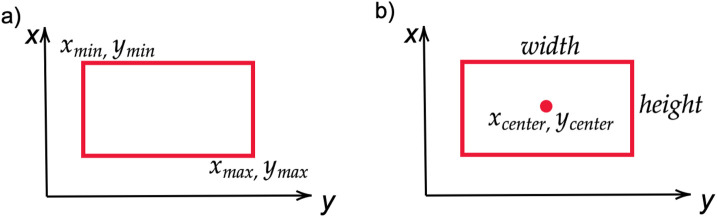
Bounding boxes in **(a)** object detectors; **(b)** YOLO detectors.

These performance metrics evolved based on prediction boxes produced by detection models. The output of object detectors is bounding boxes encoded as ( $x_{\text{min}}$ , $y_{\text{min}}$ , $x_{\text{max}}$ , $y_{\text{max}}$ ), where each pair of coordinates represent top-left and bottom-right coordinates as shown in Figure 4a. The exception is YOLO family models, in particular, YOLOv8 has a bounding box represented by (label, $x_{\text{center}}$ , $y_{\text{center}}$ , width, height), where label is the class of the object, ( $x_{\text{center}}$ , $y_{\text{center}}$ ) are normalized coordinates of the center of bounding box and (width, height) are its width and height as shown in Figure 4b (Padilla et al., 2020). Despite these differences, the final evaluation metrics, such as F1 score, AP, and mAP, remain unaffected.

**FIGURE 5:**
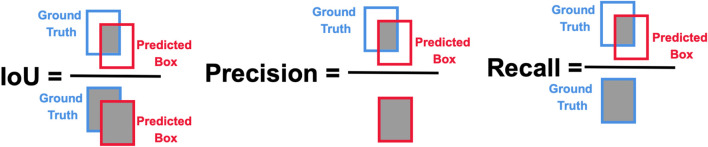
IoU, precision and recall.

The Intersection of Union (IoU) is a measure of the overlap between predicted and Ground Truth (GT) bounding boxes. Based on the given specific threshold θ , classification can be considered as correct or incorrect. In particular, if IoU is above the threshold θ , a prediction is considered a True Positive (TP). Otherwise, there are two cases of incorrect detection: False Negative (FN) and False Positive (FP). FN occurs when the object detector fails to identify an object that is present in the scene, whereas FP happens when the model incorrectly detects an object in an area where none exists. The next evaluation metrics are Precision (P) and Recall (R). Precision (P) shows the ability of the model to find only relevant objects and can be found using Equation 1, while Recall (R) measures the proportion of actual GT objects that were correctly detected and can be identified using Equation 2. Visualization of IoU, precision P and recall R is illustrated in Figure 5.

$$P=\frac{TP}{TP+FP}=\frac{TP}{\text{all detections}};$$ (1)

$$R=\frac{TP}{TP+FN}=\frac{TP}{\text{all ground truth}};$$ (2)

**FIGURE 6:**
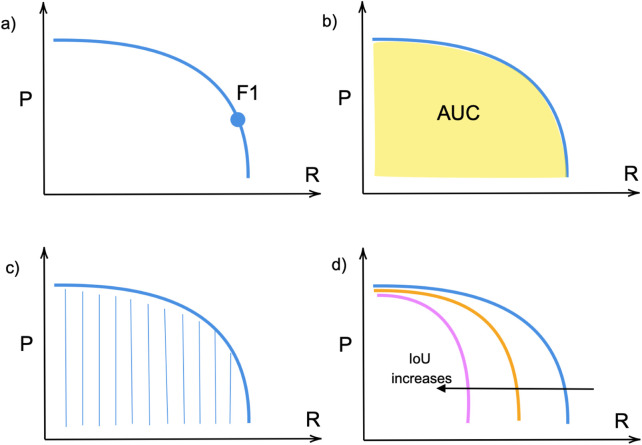
**(a)** F1 score; **(b)** Precision-Recall Area Under Curve (PR-AUC); **(c)** Average precision (AP); **(d)** mAP over various IoU.

The precision-recall curve illustrates a trade-off at various confidence values. The model is considered good if the precision remains high as its recall increases (Padilla et al., 2020). The F1 score is the metric that shows the trade-off between precision P and recall R as illustrated in Figure 6a and can be found from Equation 3. It ranges between 0 and 1, where 1 shows the highest accuracy. Average Precision (AP) is identified individually for each class and represents the area under the curve (AUC) of the precision-recall corresponding to Figure 6b for that specific class as shown in Figure 6c. It measures how well the model balances precision (accuracy of positive predictions) and recall (coverage of actual positives) at different confidence thresholds. Eventually, mAP (Figure 6d) is the average of the Average Precision (AP) of each class. ${\text{mAP}}_{50}$ is the mean average precision of a model when the IoU threshold is set to 50%, whereas ${\text{mAP}}_{50:95}$ evaluates performance across multiple IoU thresholds between 50% and 95%, and is more difficult to achieve compared to ${\text{mAP}}_{50}$ . ${\text{mAP}}_{50:95}$ is preferred metric for benchmarking state-of-the-art models.

$$\text{F}1\text{ score}=2\times \frac{P\times R}{P+R};$$ (3)

In addition to mAP, which represents the prediction quality, the number of floating point operations (FLOPs) is commonly used to measure the computational efficiency and complexity of a model (Messikommer et al., 2020). For asynchronous models, where data is event-driven rather than frame-based, the adopted metric is FLOPs per event (FLOPs/ev) (Santambrogio et al., 2024), which more accurately reflects the computational cost relative to the number of events processed.

Another important performance indicator is the runtime of the object detection model, referring to the time required to process the input data and evaluate all bounding box annotations across the images. Lower runtime is crucial, especially in real-time or resource-constrained applications such as robotics and autonomous systems.

Besides, there are evaluations such as latency (milliseconds(ms)) , throughput ( framespersecond or eventspersecond ), energy efficiency ( Joules or Watts ) and memory footprint (MB) (Iaboni and Abichandani, 2024), which better capture a model’s practical viability on neuromorphic hardware or embedded systems and contribute to the overall computational cost. Balancing accuracy, computational cost, and speed is essential for deploying efficient and scalable event-based object detection models. All the above-mentioned metrics are summarized in Table 5.

**TABLE 5:** Object detector performance evaluation metrics.

Metric	Units	Description
Intersection of Union (IoU)	unitless	Overlap between predicted and Ground Truth (GT) boxes
Precision (P)	unitless	Shows of all predicted boxes, how many were actually correct
Recall (R)	unitless	Shows of all actual objects, how many were found by model
F1 score	between 0 and 1	Summarizes the accuracy of predicted bounding boxes
Average Precision (AP)	unitless	Area under this Precision-Recall curve (per class performance)
Mean Average Precision	unitless	Average of the precision-recall curve across different IoU thresholds and/or multiple classes (overall detector performance)
Throughput	Frames per second (FPS)	Number of frames processed by model per second, speed of processing
Runtime	ms	Inference speed
Energy	Joules or Watt	Energy consumption required for inference
Memory footprint	Mega Bytes	Amount of memory a model needs to operate
Model complexity	MACs, FLOPs	Amount of computation required for inference

### 4.2 Models

**FIGURE 7:**
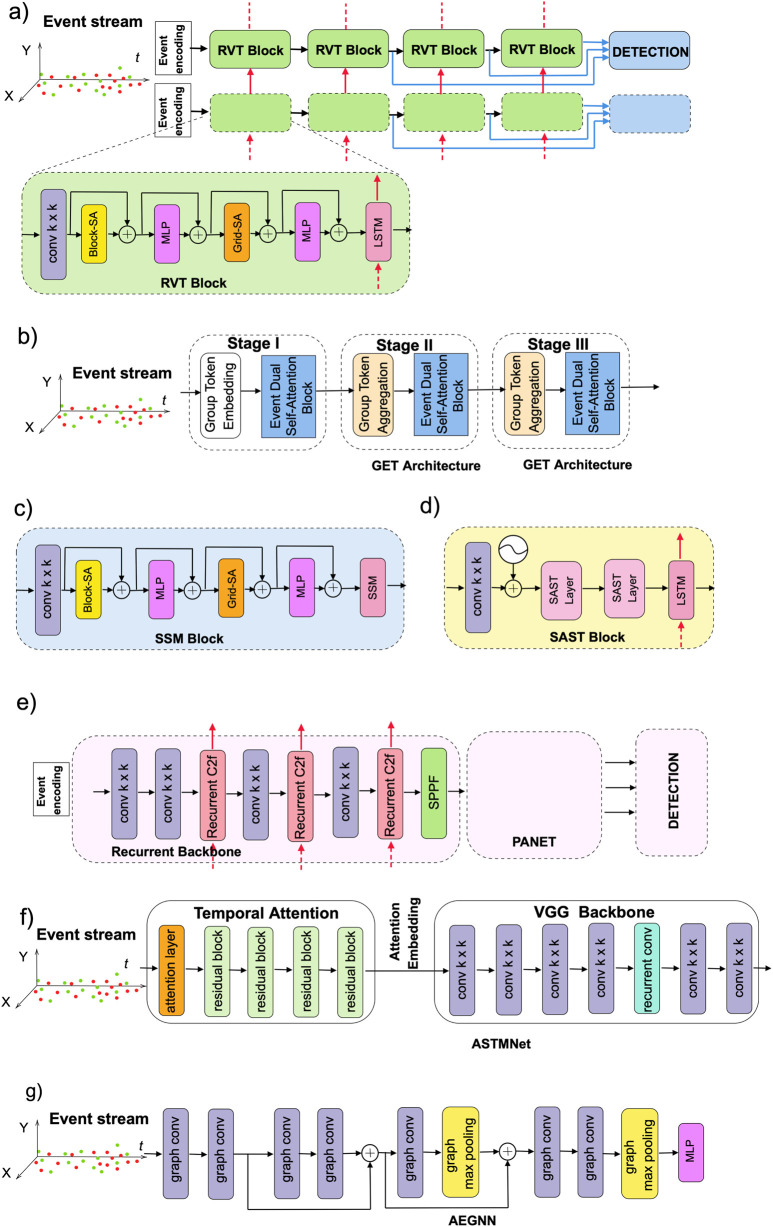
Schematic of fixed-rate **(a)** RVT and RVT Block; **(b)** GET architecture; **(c)** SSM Block; **(d)** SAST Block; **(e)** Recurrent YOLOv8; and asynchronous **(f)** ASTMNet; **(g)** AEGNN models.

As mentioned earlier, event data is a new and fundamentally different type of information compared to traditional data. Nevertheless, existing neural models have been adapted to effectively process event streams. These approaches can be broadly categorized into dense, asynchronous dense, SNNs, GNNs, and other model types. Below, we present these categories with a focus on state-of-the-art models for autonomous event-based object detection, particularly those evaluated on the GEN1, 1MP, and eTraM datasets. Figure 7 illustrates some of them.

#### 4.2.1 Dense models

Currently, DNNs remain a practical choice for event-based data processing due to their well-established training methodologies and scalability. In particular, in (Perot et al., 2020; Silva et al., 2024c; Peng et al., 2023a), authors evaluated the performance of popular CNN-based RetinaNet and YOLOv5 models on GEN1 and 1MP datasets, which lately served as a baseline for their frameworks. However, it should be noted that conventional models require event streams to be converted into a grid-like format before they can be processed. Earlier methods often relied on reconstructing grayscale images from events (Liu et al., 2023; Perot et al., 2020), while recent works use more advanced encoding techniques (Peng et al., 2023a; Liu et al., 2023; Peng et al., 2023b), which are discussed later in Section 4.3.

Generally, DNN-based backbones can be categorized into either CNN-based or Transformer-based architectures. Additionally, they can be improved by incorporating specialized architectural layers to better capture the temporal dynamics of event data. In particular, networks that integrate recurrent layers form a distinct subgroup of models. One of the first models with recurrency is Recurrent Event-camera Detector (RED) (Perot et al., 2020). The architecture of RED includes convolutional layers extracting low-level features followed by convolutional long short-term memory (ConvLSTM) layers to extract high-level spatio-temporal patterns from the input. RED showed that memory mechanism created by recurrent layers allows detection of objects directly from events, achieving results comparable to those obtained using reconstructed grayscale images. However, utilization of ConvLSTM layers also led to increased computational complexity and latency and resulted in slow inference.

The Agile Event Detector (AED) is a YOLO-based architecture, which demonstrated faster and more accurate performance than the baseline YOLOX model on the GEN1 and 1MP datasets (Liu et al., 2023). Prior to AED, many event-based detection models were computationally intensive and suffered from low inference speeds. In addition, conventional approaches for converting events into dense representations often rely on fixed global time windows $t_{w}$ , which fail to account for the different motion speeds. Specifically, long time windows can lead to motion blur for fast-moving objects, while short windows may not capture sufficient information for slower ones. AED overcomes this limitation through a specialized event encoding technique, enabling a motion-robust, high-speed, and lightweight detection pipeline. The architecture of AED avoided using recurrent layers due to the higher cost of training and slower speed during inference.

The next architecture is Recurrent Vision Transformer (RVT) (Gehrig and Scaramuzza, 2023) and has a transformer-based backbone with recurrent layers. RVT is designed to overcome a trade-off between accuracy and computational complexity of previous event-based object detectors (Perot et al., 2020; Messikommer et al., 2020). It has a hierarchical multi-stage design of several blocks, which include an attention mechanism to process spatio-temporal data. Moreover, to reduce computation, RVT blocks gave preference to Vanilla LSTM cells over ConvLSTM layers, which allowed for a decrease in inference time compared to the RED. Following the introduction of RVT, numerous event-based object detection models were proposed within a relatively short period, and RVT served as a baseline for the majority of them, as can be noticed below.

In most cases, converting events to an image-like dense format can result in the loss of some properties. A group-based vision Transformer backbone called Group Event Transformer (GET) tried to overcome this problem by incorporating Group Token representation of asynchronous events that consider their time and polarity (Peng et al., 2023b). The architecture of GET has three stages comprised of Group Token Embedding (GTE), Event Dual Self-Attention (EDSA), and Group Token Aggregation (GTA) blocks. The visualization study demonstrated that by incorporating the EDSA block, GET could effectively capture counterclockwise motion. The enhanced version of GET with ConvLSTM layers was able to outperform most state-of-the-art models like RED, RVT-B, and others. Overall, GET is reported to be the fastest end-to-end method since other frameworks require longer data pre-processing time, which is typically not omitted in runtime results.

Traditional Vision Transformers benefit from the self-attention mechanism, which improves performance by capturing long-range dependencies. However, its quadratic computational complexity also introduces a great overhead in terms of A-FLOPs (Attention-related FLOPs) and limits scalability during processing high-resolution tasks (Gehrig and Scaramuzza, 2023; Peng et al., 2024). One of the ways to reduce computational burden was using sparse and sparse window-based transformers that rely on token-level sparsification or adaptive sparsification. In the event-based domain, these ideas were implemented in the Scene adaptive sparse transformer (SAST) (Peng et al., 2024). Its architecture is composed of multiple SAST blocks, each of which concludes with an LSTM layer. Through the combined use of window-token co-sparsification and Masked Sparse Window Self-Attention (MS-WSA), SAST effectively discards uninformative windows and tokens. This enables scene-aware adaptability, which allows focusing only on relevant objects. As a result, it could achieve better performance than variants of RVT at lower computational expense.

Recurrent YOLOv8 (ReYOLOv8) is an object detection framework that leverages the state-of-the-art CNN-based YOLOv8 model for efficient and fast object detection, and enhances its spatiotemporal processing capabilities to process events by integrating ConvLSTM layers (Silva et al., 2025). ReYOLOv8 achieved better accuracy with a relatively smaller number of parameters compared to other state-of-the-art event-based object detectors, including RED (Perot et al., 2020), GET (Peng et al., 2023b), SAST (Peng et al., 2024), variants of RVT (Gehrig and Scaramuzza, 2023), HMNet (Hamaguchi et al., 2023), and others.

As mentioned earlier, prior to being processed by dense models, the event stream must be converted into a frame-like format. The time window $t_{w}$ used to generate dense event representations may vary between training and inference. When models are unable to adapt to these differences in frequency, their detection performance can degrade. Integration of the State Space Model (SSM), a type of model designed to handle sequential data efficiently over long time horizons, may improve their performance without retraining at different frequencies (Zubic et al., 2024). Evaluation of RVT and SSM-ViT represented by SSM for Sequence Modeling (S4) (Gu A. et al., 2021), Diagonal S4 (S4D) (Gu et al., 2022), and SSM with parallel scans (S5) (Smith et al., 2022) models across different frequencies showed that SSM-ViT can outperform RVT by 20 mAP and a 33% increase in training speed (Zubic et al., 2024).

SSM with 2D selective scan (S6) was adopted in the architecture of Sparse Mamba (SMamba) (Yang et al., 2025). It was evaluated on widely adopted GEN1, 1MP datasets and the recent eTRaM dataset, and outperformed the state-of-the-art models, including its sparse transformer-based counterpart SAST. While SAST proposed a window attention-based sparsification strategy, SMamba utilizes information-guided spatial selective scanning and global spatial-based channel selective scanning that can measure the information content of tokens and discard non-event noisy tokens.

#### 4.2.2 Asynchronous dense models

Conversion of a stream of asynchronous and spatially sparse events into a synchronous tensor-like format and processing them by dense models at fixed rates leads to high latency and computational costs. Therefore, some works focus on dense models that process asynchronous event-by-event data during inference, leveraging both the temporal and spatial features of the event information. Nevertheless, training asynchronous dense models still requires converting raw event data into frame-like representations, which remains computationally intensive.

AsyNet is a framework designed to convert traditional models, trained on synchronous dense images, into asynchronous models that produce identical outputs (Messikommer et al., 2020). To preserve sparsity in event-based input data, AsyNet employs a sparse convolutional (SparseConv) technique such as the Submanifold Sparse Convolutional (SSC) Network, which effectively ignores zero-valued inputs within the convolutional receptive field. To maintain temporal sparsity, Sparse Recursive Representations (SRRs) are used. Unlike traditional methods that reprocess the entire image-like representation from scratch for every incoming event, SRRs enable recursive and sparse updates as new events arrive, which eliminates the need to rebuild the full representation each time. Examples of SRRs include event histograms (Maqueda et al., 2018), event queues (Tulyakov et al., 2019), and time images (Mitrokhin et al., 2018), where only single pixels need updating for each new event.

The next approach for asynchronous processing is known as MatrixLSTM and uses a grid of Long Short-Term Memory (LSTM) cells to convert asynchronous streams of events into 2D event representations (Cannici et al., 2020). All outputs of LSTM layers are collected into a dense tensor of shape H×W×C , forming a final surface $S_{\varepsilon }$ . By jointly training MatrixLSTM layers with state-of-the-art models, there is no longer a need for pre-processing events into a frame-like structure to process the input.

Asynchronous spatio-temporal memory network for continuous event-based object detection (ASTMNet) also processes raw event sequence directly without converting to image-like format (Li J. et al., 2022). This became possible due to the utilization of an adaptive temporal sampling strategy and temporal attention convolutional module.

Fully Asynchronous, Recurrent and Sparse Event-based CNN (FARSE-CNN) uses hierarchical recurrent units in a convolutional way to process sparse and asynchronous input (Santambrogio et al., 2024). Unlike MatrixLSTM, which also uses ConvLSTM but uses a single recurrent layer, FARSE-CNN is a multi-layered hierarchical network. FARSE-CNN also introduced Temporal Dropout, a temporal compression mechanism, which allows building deep networks.

The transformer-based framework for streaming object detection (SODformer) also operates asynchronously without being tied to a fixed frame rate (Li et al., 2023). SODformer was designed for object detection based on heterogeneous data, and, to improve detection accuracy from event- and frame-based streams, it introduced transformer and asynchronous attention-based fusion modules. The performance of SODformer was evaluated on the multimodal PKU-DAVIS-SOD dataset.

#### 4.2.3 Spiking Neural Networks

As observed in dense models, adding recurrent connections can enhance the performance of dense backbones due to the ability to capture the temporal dependencies of events (Perot et al., 2020; Gehrig and Scaramuzza, 2023). One study further showed that Spiking Neural Networks (SNNs) outperform standard RNNs in processing sparse, event-driven data and achieve performance comparable to LSTMs (He et al., 2020). SNNs are widely known as biologically inspired, energy-efficient architectures that are inherently well-suited for processing asynchronous input (Cordone et al., 2022) and are considered as neuromorphic or/and event-driven neural networks. However, as the resolution of the vision data increases, the performance of SNNs begins to decline (He et al., 2020). Moreover, SNNs face significant challenges when it comes to training and scalability, primarily due to their inherent complexity and the need for algorithms to handle the discrete and event-driven nature of their neurons (Kim et al., 2020). Besides, there is a lack of specialized hardware. Traditional gradient-based training methods and Graphics Processing Units (GPUs) and Tensor Processing Units (TPUs) are well-optimized for DNNs, but not directly suitable for SNNs (Cordone, 2022). Different topologies of SNNs and training methods are continuously evolving. Additionally, pre-trained DNNs can be converted into SNNs for inference, often achieving results comparable to those obtained with DNNs (Silva D. et al., 2024).

One of the first spike-based object detection models is a Spiking-YOLO, which was obtained via DNN-to-SNN conversion (Kim et al., 2020). Initially, the converted model was unable to detect any objects due to a low firing rate and a lack of an efficient implementation method of leaky-ReLU. After introducing channel-wise normalization and signed neurons with an imbalanced threshold, the modified model achieved up to 98% on non-trivial PASCAL VOC and MS COCO datasets, comparable to the original DNN-based TinyYOLO model. However, applied normalization methods also led to an increase in the required number of timesteps, which is unfeasible for real-world implementation on neuromorphic hardware due to high latency (Cordone, 2022). In particular, the conversion-based Spiking-YOLO model (Kim et al., 2020) required 500 timesteps to achieve results comparable to those of the Trainable Spiking-YOLO (Tr-Spiking-YOLO) (Yuan et al., 2024), which uses direct training with the surrogate gradient algorithm and only 5 timesteps on the GEN1 dataset.

EMS-YOLO is the first deep spiking object detector trained directly with surrogate gradients, without relying on ANN-to-SNN conversion Su et al. (2023). EMS-YOLO uses the standard Leaky Integrate-and-Fire (LIF) neuron model and surrogate gradient backpropagation through time (BPTT) across all spiking layers. On the GEN1 dataset, EMS-ResNet10 achieves performance comparable to dense ResNet10 while consuming 5.83 × less energy.

End-to-End Adaptive Sampling and Representation for Event-based Detection with Recurrent Spiking Neural Networks (EAS-SNN) is another SNN-based model that introduced Residual Potential Dropout (RPD) and Spike-Aware Training (SAT) (Wang Z. et al., 2024). It also uses backpropagation through time (BPTT) with surrogate gradient functions to overcome the non-differentiability of spikes. Surrogate gradient applied in Spike-Aware Training (SAT) improves the precision of spike timing updates. With only 3 timesteps required for detection, EAS-SNN demonstrated competitive detection speeds of 54.35 FPS and reduced energy consumption up to a 5.85×.

A recently introduced Multi-Synaptic Firing (MSF) neuron inspired by multisynaptic connections represents a practical breakthrough for event-based object detection Fan et al. (2025). Unlike vanilla spiking neuron, MSF-based SNN is capable of simultaneously encoding spatial intensity through firing rates and temporal dynamics through spike timing. By combining multi-threshold and multi-synaptic firing with surrogate gradients, MSF networks can be trained at scale for deep model architectures. Particularly, the MHSANet-YOLO model with MSF neurons achieved up to 73.7 mAP on the GEN1 dataset, which is better than both ReLU and LIF versions. Moreover, MSF-based MHSANet-YOLO required 16.6 × less power consumption than the one with ReLU neurons Fan et al. (2025).

#### 4.2.4 Graph-based models

The architecture of GNNs can also process event-based data by preserving their sparsity and asynchronous nature. One of the GNN-based object detection frameworks, called Asynchronous Event-based Graph Neural Network (AEGNN) processes events as “static” spatio-temporal graphs in a sequential manner (Schaefer et al., 2022). AEGNN uses an efficient training method where only the affected nodes are updated when a single event occurs. In other words, they were able to process events sparsely and asynchronously. In addition, it can also process batches of events and use the standard backpropagation method. This enables AEGNN to be trained on synchronized event data and support asynchronous inference. For object detection tasks, AEGNN demonstrated up to 200 × less computational complexity.

The asynchronous nature of the event stream is also considered in Efficient Asynchronous Graph Neural Networks (EAGR) (Gehrig and Scaramuzza, 2022). EAGR offers per-event processing and can be configured using several architecture design choices. To reduce computational cost, it used max pooling in early layers and a pruning method, which resulted in skipping up to 73% of node updates. Therefore, a reduced number of FLOPS was observed during the first three layers while processing GEN1 dataset. A small size variant of EAGR achieved a 14.1 mAP higher performance and around 13% times fewer MFLOPS/ev than the AEGNN. Nevertheless, GNN-based models’ performance is still behind dense counterparts, especially involving recurrent connections.

Deep Asynchronous GNN (DAGr) attempted to improve GNN’s performance by combining event- and frame-based sensors in a hybrid object detector (Gehrig and Scaramuzza, 2024). The study showed that combining a 20-FPS RGB camera with high-rate event cameras can match the latency of a 5000-FPS camera and the bandwidth of a 45-FPS camera. Similarly to EAGR, it comes with different variants of configurations, conditionally divided into nano, small, and large size models. By effectively leveraging each modality, the large variant of DAGr achieved improved performance, reaching 41.9 mAP by the large size variant.

#### 4.2.5 Other models

Some architectures cannot be categorized into the aforementioned groups and include frameworks that are employed to enhance the performance of the object detectors.

The first one is Hierarchical Neural Memory Network (HMNet) (Hamaguchi et al., 2023). It is a multi-rate network architecture inspired by Hierarchical Temporal Memory (HTM). An ordinary HTM is a brain-inspired algorithm that uses an unsupervised Hebbian-learning rule and is characterized by sparsity, hierarchy, and modularity. It operates at a single rate and incorporates Spatial Pooling and Temporal Pooling acting as convolutional and recurrent layers (Smagulova et al., 2019). On the other hand, HMNet features a temporal hierarchy of multi-level latent memories that operate at different rates, allowing it to capture scenes with varying motion speeds (Hamaguchi et al., 2023). In HMNet, low-level memories encode local and dynamic information, while high-level memories focus on static information. For embedding the sparse event stream into dense memory cells, HMNet introduced an Event Sparse Cross Attention (ESCA). There are four variants of HMNet, including HMNet-B1/L1/B3/L3, which differ in the number of memory levels and dimensions. In addition, the architecture of HMNet can be extended to the multisensory inputs. Overall, HMNet outperforms other methods in speed, particularly the recurrent baselines, which require a long accumulation time to construct an event frame.

The dense-to-sparse event-based object detection framework, DTSDNet, provides enhanced speed robustness and enables a reduction in event stream accumulation time by a factor of five, such as decreasing it from the typical 50 ms to just 10 ms (Fan et al., 2024). In particular, in conventional recurrent models, event streams are partitioned evenly, whereas DTSDNet uses an attention-based dual-pathway aggregation module to integrate rich spatial information from dense pathway with asynchronous sparse pathway.

While manually designed architectures like HMNet and others demonstrate strong performance, they often rely on expert knowledge and trial-and-error. To overcome this limitation and explore more efficient configurations, Neural Architecture Search (NAS) can automate the design of novel neural networks by exploring various combinations of architectural components using strategies like gradient-based search, evolutionary algorithms, and reinforcement learning (Ren et al., 2021). Chimera is the first block-based Neural Architecture Search (NAS) for event-based object detection using dense models (Silva et al., 2024b). The choice of encoding format, along with models designed using the Chimera NAS framework, achieved performance comparable to state-of-the-art models on the GEN1 and PEDRo datasets, while reducing the number of parameters up to 1.6×.

There are also hybrid models that include both SNN and dense Artificial neural network (ANN) architectures. One of such examples is an attention-based hybrid SNN-ANN. Its SNN part captures spatio-temporal events and converts them into dense feature maps to be further processed by the ANN part (Ahmed et al., 2025). SNN component of Hybrid SNN-ANN model used the surrogate gradient approach during training. Hybrid SNN-ANN achieves dense-like performance at a reduced number of parameters, latency, and power.

### 4.3 Event encoding techniques

Each event in a event stream E occurs only due to the change in the captured scene and can be recorded in a sequence $e_{k}$ = $\left(x_{k},y_{k},t_{k},p_{k}\right)$ of k = 1, 2, … N events, where (x,y) represent pixel location, t is the time and p is the polarity. In a 4-dimensional manifold of x,y,t,p , a point-set of events can be represented as an event field, a continuous time representation of events of positive and negative polarity $E_{+}$ and $E_{-}$ as in Equation 4:

$$S_{\pm }\left(x,y,t\right)=\underset{e_{k}\in E_{\pm }}{\sum }\delta \left(x-x_{k},y-y_{k}\right)\delta \left(t-t_{k}\right)$$ (4)

SNNs are inherently suited for processing event-based data. Models that utilize asynchronous sparse architectures are also capable of handling raw events. However, in the case of DNNs and GNNs, events cannot be processed directly by models and need to be encoded into a specific format. To be utilized by GNNs, events must first be transformed into a graph format (Gehrig and Scaramuzza, 2022; 2024), whereas DNNs process events that have been adapted into the image- or tensor-like structure.

During event encoding into a specific format, the choice of representation can significantly impact performance. For example, the temporal component of the event stream can be used to identify patterns and provide valuable insights in certain applications, a concept known as temporal sensitivity (Shariff et al., 2024). Additionally, focusing on the most informative changes in a scene, which is called selectivity, further improves processing. These representations can also be used to satisfy computational and memory requirements (Shariff et al., 2024). Table 6 presents a summary of common event encoding formats, with detailed descriptions provided in the sections below.

**TABLE 6:** Common event encoding techniques [adapted from (Gehrig et al., 2019; Zheng et al., 2023].

Type	Event representation	Dimension	Description
Dense	Event frame (Henri et al., 2017)	H × W	Event stream is divided into two polarities ON and OFF, forming a two-channel image and is then combined to create an event frame Discards temporal and polarity information
Dense	Event count image (Zhu et al., 2018b)	2 × H × W	Discards time stamps
Dense	Surface of Active Events (SAE) (Zhu et al., 2018b)	2 × H × W	Discards earlier time stamps
Dense	Voxel grid (Zhu et al., 2019)	H × W × T	Discards event polarity
Dense	Voxel Cube (Cordone et al., 2022)	C × T × H × W	Event stream is divided into multiple n temporal bins and events split into channels C
Dense	Histogram of Time Surfaces (HATS) (Sironi et al., 2018)	2 × H × W	Discards temporal information
Dense	Event Spike Tensor (EST) (Gehrig et al., 2019)	2 × B × H × W	Discards the least amount of information
Dense	Temporal Active Focus (TAF) (Liu et al., 2023)	2 K × H × W	A dense version of EST that samples only recent non-zero event
Dense	Mixed-Density Event Stacks (MDES) (Nam et al., 2022)	M × C × H × W	Selects the most recent events within the time window and aggregates event sequences into multiple stacks M with varying densities to better capture objects moving at different speeds
Dense	Stacked Histogram (SHIST) (Gehrig and Scaramuzza, 2023)	2B × H × W	Event stream is divided into multiple temporal bins and events split into two polarities ON and OFF, forming a structured spatiotemporal tensor that preserves motion and polarity information
Dense	Volume of Ternary Event Images (VTEI) (Silva et al., 2025)	B × H × W	Event stream is divided into multiple temporal bins, and for each bin, the most recent events are sampled to generate a Ternary Event Image (TEI). Stacking the TEIs from all bins results in a Volume of Ternary Event Images (VTEI), capturing both spatial and temporal structure
Dense	Group Token (Peng et al., 2023b)	2 K × H × W	Event stream is divided into K intervals and events are mapped to patches with own rank and position
Dense	Time-Ordered Recent Event (TORE) (Baldwin et al., 2022)	2 K × H × W	Time-ordered recent event volumes
Dense	12-channel Event Representation through Gromov-Wasserstein Optimization (ERGO-12) (Zubić et al., 2023)	C × H × W	Event representation from GWD optimization (measures the distortion rate from raw events to event representation)
Graph	Graph (Gehrig and Scaramuzza, 2022; Gehrig and Scaramuzza, 2024)	n/a	The graph that include information about spatial and temporal position of the event
Spike	Spike (Wang Z et al., 2024b; Ahmed et al., 2024)	n/a	To reduce temporal resolution of event stream a sampling S and aggregation A techniques might be adopted

#### 4.3.1 Dense aggregation

**FIGURE 8:**
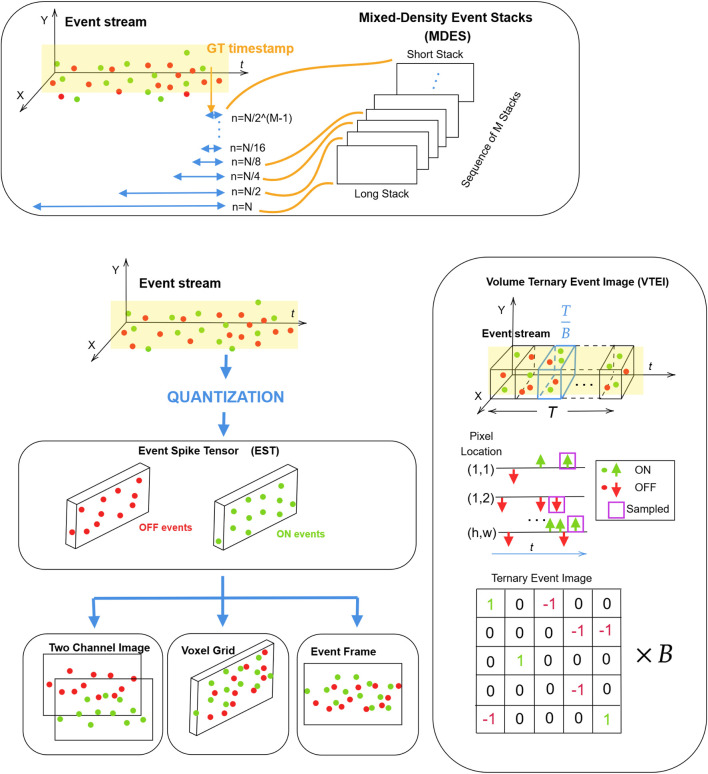
Some of the dense representations of events: Event Spike Tensor (EST), Two-Channel Image, Voxel Grid, Event Frame, Mixed-Density Event Stacks (MDES), Volume Ternary Event Image (VTEI) (adapted from (Gehrig et al., 2019; Nam et al., 2022; Silva et al., 2025).

A common approach for converting an event stream into a dense, grid-like format involves stacking the events in various configurations. Based on image formation strategies, existing stacking methods are categorized into four types: stacking by polarity, timestamps, event count, and a combination of timestamps and polarity (Zheng et al., 2023). This section highlights several noteworthy techniques for encoding events and illustrates some of them in Figure 8.

- Event Frame is formed by merging two-channel images, each corresponding to stacked ON and OFF polarity events (Henri et al., 2017).
- Event Volume or Voxel Grid is a volumetric representation of the events expressed as (H,W,T) (Zhu et al., 2019). An event stream containing N events within a global range ( $t_{0}$ , $t_{N}$ ) is sampled into the T temporal bins ranging between [0, B−1 ] with a normalized timestamp $t_{k}^{*}$ as in Equation 5:

$$t_{k}^{*}=\frac{\left(t_{k}-t_{0}\right)}{\left(t_{N}-t_{1}\right)}T$$ (5)

Each element in the event volume consists of events represented by a linearly weighted accumulation, analogous to bilinear interpolation as in Equation 6:

$$V\left(x,y,t\right)=\underset{k}{\sum }p_{k}k_{b}\left(x-x_{k}\right)k_{b}\left(y-y_{k}\right)k_{b}\left(t-t_{k}^{*}\right)$$ (6)

where $k_{b}\left(a\right)$ = max(0, 1- |a| ) is a bilinear kernel ensuring smooth interpolation across the discretized space (Jaderberg et al., 2015).

- Voxel Cube are obtained from a voxel grid which is formed via accumulation of events over a specified time window Δt (Cordone et al., 2022). In particular, a sample lasting d seconds would be divided into T = d / Δt timesteps. The resulting voxel grid is stored in 4 DCTHW format, where C is the number of channels, T denotes the number of timesteps, also known as bins, and H and W correspond to the height and width of data, respectively. Voxel Cubes are obtained by further dividing Δt into micro time bins.
- Event Spike Tensor (EST) allows to process continuous-time event data as a grid-like 4-dimensional data structure (2T,H,W) (Gehrig and Scaramuzza, 2024). Event stream is converted to EST through a sequence of differentiable operations: kernel convolutions, quantizations, and projections. Generalized EST that retains all four dimensions that can be used to derive new and existing representations.

In a given time interval Δτ , events represent point-sets that can be summarized by the *event field*, which can be interpreted as successive measurements of a function $f_{\pm }$ or the *Event Measurement Field* (EMF) according to Equation 7:

$$S_{\pm }\left(x,y,t\right)=\underset{e_{k}\in E_{\pm }}{\sum }f_{\pm }\left(x,y,t\right)\delta \left(x-x_{k},y-y_{k}\right)\delta \left(t-t_{k}\right)$$ (7)

Examples of $f_{\pm }$ include event polarity (e.g., $f_{\pm }\left(x,y,t\right)=\pm$ 1), event count (e.g., $f_{\pm }\left(x,y,t\right)=$ 1) and the normalized time stamp (e.g., $f_{\pm }\left(x,y,t\right)=\frac{t-t_{0}}{\Delta t}$ ). Since events are modeled as a Dirac pulse δ and are difficult to use directly, EMF is convolved with a kernel k(x,y,t) to aggregate and smooth the events as in Equation 8:

$$\left(k*S_{\pm }\right)\left(x,y,t\right)=\underset{e_{k}\in E_{\pm }}{\sum }f_{\pm }\left(x_{k},y_{k},t_{k}\right)k\left(x-x_{k},y-y_{k}\right)\delta \left(t-t_{k}\right)$$ (8)

The convolved signal is also known as *membrane potential*. Prior works employed various task-specific kernel functions, including the exponential kernel, which was used in the hierarchy of time-surfaces (HOTS) (Lagorce et al., 2016) and histogram of average time surfaces (HATS) (Sironi et al., 2018) encodings. After a convolutional step, the signal is further sampled at regular intervals to produce a grid-like generalized Event Spike Tensor (EST) representation as in Equation 9:

$$S_{\pm }\left[x_{l},y_{m},t_{n}\right]=\left(k*S_{\pm }\right)\left(x_{l},y_{m},t_{n}\right)=\underset{e_{k}\in E_{\pm }}{\sum }f_{\pm }\left(x,y,t\right)\delta \left(x_{l}-x_{k},y_{m}-y_{k}\right)\delta \left(t_{n}-t_{k}\right)$$ (9)

with the spatiotemporal coordinates $x_{l},y_{m},t_{n}$ belonging to a voxel grid (H,W,T) : $x_{l}\in$ {0,1,… W−1 }, $y_{m}\in$ {0,1,… H−1 } and $t_{n}\in$ { $t_{0},t_{0}+\Delta t$ , … $t_{0}$ + TΔt }, where $t_{0}$ is the first timestamp, Δt is the bin’s size and T is the number of temporal bins.

The generalized EST can be further modified via different operations such as summation ∑ , maximization max , and others, that can be expressed as the projection operator $H_{v}$ with v denoting dimension. EST without projection is $S_{\pm }\left[x_{l},y_{m},t_{n}\right]$ . The projection operator $H_{v}$ applied to EST $S_{\pm }\left[x_{l},y_{m},t_{n}\right]$ can result in other representations such as Event Frame $S\left[x_{l},y_{m}\right]$ = $H_{t_{n},\pm }\left(S_{\pm }\left[x_{l},y_{m},t_{n}\right]\right)$ (Henri et al., 2017), Two-Channel Image $S_{\pm }\left[x_{l},y_{m}\right]$ = $H_{t_{n}}\left(S_{\pm }\left[x_{l},y_{m},t_{n}\right]\right)$ (Maqueda et al., 2018) and Voxel Grid $S\left[x_{l},y_{m},t_{n}\right]$ = $H_{\pm }\left(S_{\pm }\left[x_{l},y_{m},t_{n}\right]\right)$ (Zhu et al., 2019).

- Temporal Active Focus (TAF) is seen as a dense version of the Event Spike Tensor (EST), which involves spatiotemporal data processing with efficient queue-based storage (Liu et al., 2023). While traditional EST is a sparse tensor covering the entire event stream E and requiring high time and storage costs, TAF focuses on sampling only the most recent non-zero K events at each spatial and polar position and thus avoids excessive data processing. Moreover, since object detection on the event stream occurs every Δτ sampling period, the TAF tensor can be incrementally updated using a First-In-First-Out (FIFO) queue to reduce computational overhead. Eventually, FIFO sliding queues of events FIFO(p,t,x,y) with depth K form a compact and dense tensor S $\in \;R^{2K\times H\times W}$ of most meaningful data. The process of TAF tensor formation is similar to EST and includes measurement function f(⋅) and convolutional kernel k(⋅) components. Here, a rectangular window function acts as a convolution kernel to detect which events contribute to the tensor. To preserve the absolute position information on the temporal dimension, the measurement function f(⋅) calculates the average elapsed time from the events captured by the convolution kernel to the current detection time $t^{\left(n\right)}$ .

According to Equation 10, at each detection step n , the average time elapsed is calculated:

$$\Delta t^{\left(n\right)}\left(E,t,x,y,p\right)≔\underset{e_{k}\in E}{\sum }f\left(x_{k},y_{k},p_{k},t_{k},t^{\left(n\right)}\right)k\left(x-x_{k},y-y_{k},p-p_{k},t-t_{k}\right)$$ (10)

Then its non-zero values are pushed into the FIFO queues. At the next step n+1 , new values are determined and pushed, while old ones are incrementally updated: $t^{\left(n+1\right)}\leftarrow t^{\left(n\right)}+\Delta \tau$ . Logarithmic transformations are applied to normalize Δt values. A dense TAF tensor is generated by continuous updates of the FIFO queues and transformations. Such incremental updates reduce the computational costs.

- Mixed-Density Event Stacks (MDES) was proposed to alleviate the event missing or overriding issues due to different speeds of the captured objects (Nam et al., 2022).

Due to the different speeds of the moving objects, stacking events with the pre-defined number of events or time period may lead to the loss of information. For example, short stacks can not track slow objects, whereas long stacks with excessive events may overwrite earlier scenes. To overcome the problem, Mixed-Density Event Stacks (MDES) format is proposed, where the length of each event sequence $e_{k}$ is aggregated to M = 10 stacks with a different number of events per stack (Nam et al., 2022). For M = 1 the event sequence $e_{1}$ has N = 5 million events, which linearly depend on the resolution of the camera and include all movements for a given time span. The next event sequence $e_{2}$ ends at the same ground-truth (GT) depth timestamp of $e_{1}$ but has twice less events n=N/2 . Slicing and stacking half of the events from the previous stack continues in the subsequent event sequence $e_{3}$ - $e_{M}$ and reaching the final M with $n=N/2^{\left(M-1\right)}$ .

- Stacked Histogram (SHIST) A Stacked Histogram (SHIST) is designed to save memory and bandwidth (Gehrig and Scaramuzza, 2023). The algorithm creating SHIST includes several steps. It starts by creating a 4-dimensional byte tensor. The first two dimensions are polarity and B discretization steps of time, whereas the last two are the height H and width W of the camera. For a time window [ $t_{a}$ , $t_{b}$ ), the set of events E can be represented as in Equation 11:

$$E=E\left(p,\tau ,x,y\right)=\underset{e_{k}\in E}{\sum }\delta \left(p-p_{k}\right)\delta \left(x-x_{k},y-y_{k}\right)\delta \left(\tau -\tau_{k}\right)$$ (11)

where $\tau_{k}$ = $\frac{\left(t_{k}-t_{a}\right)}{\left(t_{b}-t_{a}\right)}B$ . Then, the polarity and time in the resulting B 2-dimensional frames are flattened to a 3-dimensional shape (2 B , H , W ).

- Volume of Ternary Event Images (VTEI) Volume of Ternary Event Images (VTEI) method ensures high sparsity, low memory usage, low bandwidth, and low latency (Silva et al., 2025). Similar to MDES, VTEI focuses on the encoding of the last event data, but with uniform temporal bin sizes and considering events’ polarity, +1 and −1. The VTEI tensor is created in several steps. The first step involves the initialization of a tensor I with dimension B×H×W , where B is the number of temporal bins and H and W are the height and width of the camera. Then, an event stream with N events is sampled at a consistent time window [ $t_{a}$ , $t_{b}$ ) according to Equation 12:

$$T_{k}=\frac{\left(t_{k}-t_{a}\right)}{\left(t_{b}-t_{a}\right)}B$$ (12)

where $t_{a}$ and $t_{b}$ are the initial and final timestamps; $T_{k}$ is the temporal bin assigned for the timestamp $t_{k}$ .

- Group Token representation groups asynchronous events considering their timestamps and polarities (Peng et al., 2023b). Conversion of the event stream into GT format is done using Group Token Embedding (GTE) module. First, asynchronous time events are discretized into K intervals with time bin $\vec{d_{t}}$ and the resolution H×W is divided into P×P patches. When each event is mapped to a patch, it is assigned a patch rank $\vec{pr}$ and a location position within that patch $\vec{pos}$ . Then, arrays of $\left(\vec{d_{t}},\vec{pr},\vec{pos}\right)$ and polarity $\vec{p}$ are mapped into a signle 1D array as in Equation 13:

$$\vec{l}=\left(K\cdot H\cdot W\right)\cdot \vec{p}+\left(H\cdot W\right)\cdot \vec{d_{t}}+\frac{H\cdot W}{P^{2}}\cdot \vec{pr}++\vec{pos}$$ (13)

where:

$$\left\{\begin{array}{c} \vec{dt}=\left\lfloor K\times \frac{\vec{t}-t_{0}}{t_{\mathrm{end}}-t_{0}+1}\right\rfloor ,\\ \vec{pr}=\left\lfloor \left(\vec{x}\;\mathrm{mod}\;P\right)+\left(\vec{y}\;\mathrm{mod}\;P\right)\right\rfloor \times P\\ \vec{pos}=\left\lfloor \vec{x}/P\right\rfloor +\left\lfloor \vec{y}/P\right\rfloor \times W/P \end{array}\right.$$

Then, two 1D arrays with length H⋅W⋅2K are created via applying 1D bin count operation with weights of $\vec{l}$ and relative time $\vec{t}-\vec{t_{0}}/\vec{t_{\mathrm{end}}-t_{0}}$ . After concatenation, reshaping and 3×3 convolution operations the Group Tokens with dimension ( $\frac{H}{p}\cdot \frac{W}{P}\times \left(G\cdot C\right)$ are generated, where C is the channel number of each group and G is the number of groups and depends on combinations of time intervals and polarity.

- Time-Ordered Recent Event (TORE) volumes avoid fixed and predefined frame rates, which helps to minimize information loss (Baldwin et al., 2022). Similar to TAF, TORE prioritizes the most recent events since they have the most impact and employs FIFO buffer. TORE volumes are implemented based on a per pixel polarity specific FIFO queues FIFO(x,y,p,k) of depth k∈1,2,3..K . Each queue is the result of adding a new event and removing the oldest. According to Equation 14, TORE volume compactly stores raw spike timing information using a log-time difference between the current time t and the k most recent events in FIFO:

$$TORE\left(x,y,p,k,t\right)=max\left(min\left(log\left(t-FIFO\left(x,y,p,k\right)+1\right),log\left(\tau \right)\right),log\left(\tau^{\prime }\right)\right)$$ (14)

where τ is the maximum time and $\tau^{\prime }$ is the minimum time sensitivity. τ is optional and can be used to establish a hard threshold for memory retention, which is beneficial in scenarios with limited bandwidth. Meanwhile, $\tau^{\prime }$ helps to suppress noise amplified by the logarithm. TORE volume does not require temporal binning and windowing and can be created for any time period in the format 2K×H×W .

- 12-channel Event Representation through Gromov-Wasserstein Optimization (ERGO-12) It was discovered that several measures can improve model convergence and speed up optimization, and include (i) normalization of the event coordinates and timestamps, (ii) concatenation of the normalized pixels, and (iii) sparsification (Zubić et al., 2023).

The choice of encoding format depends on the specific task, dataset, and network backbone used. Traditionally, identifying the optimal representation relies on validation scores obtained through neural networks, which is often a resource-intensive process. A recently introduced method for ranking event representations across various formats leverages the Gromov-Wasserstein Discrepancy (GWD), achieving a 200 × speedup compared to traditional neural network-based approaches (Zubić et al., 2023). $GWD_{N}$ over N samples is an average distortion rate between raw events E and their encoded features F and correlates with neural network output according to Equation 15:

$$GWD_{N}=\frac{1}{N}\underset{i}{\sum }L\left(E_{i},F_{i}\right)$$ (15)

where $L\left(E_{i},F_{i}\right)$ is the Gromov-Wasserstein Discrepancy or the optimal cost of matching events to features under an optimal transport plan.

The tests of the two-channel 2D Event Histogram and 12-channel Voxel Grid, MDES, TORE, and ERGO-12 using YOLOv6 architecture preserved the same ranking across multiple backbones, SwinV2 (Liu et al., 2022), ResNet-50 (He et al., 2016), and EfficientRep (Weng et al., 2023). Moreover, ERGO-12 outperformed other methods by up to 2.9% mAP on the GEN1 dataset using YOLOv6 with SwinV2 backbone (Zubić et al., 2023).

#### 4.3.2 Spike-based representation

Although SNNs can naturally perform event-driven computations, their performance lags behind DNNs. One of the possible reasons is that the temporal resolution of sensors exceeds the processing capability of object detectors. Inspired by a sampling S and aggregation A mechanism used to convert events to dense tensor formats, a recent work proposed an Adaptive Sampling technique with Recurrent Spiking Neural Networks (ARSNN) and was used with the EAS-SNN model (Wang Z. et al., 2024).

#### 4.3.3 Graph representation

In AEGNN, the event stream is converted into a spatio-temporal graph format using uniform subsampling (Schaefer et al., 2022). In particular, events are embedded into a spatio-temporal space $R^{3}$ and divided into K subsamples (e.g., K = 10). During pre-processing, more informative events and their precise time are kept, whereas removed events reduce the chances of overfitting. As a result, the temporal position of each event is normalized by a factor β and each event is mapped to a node to form a graph G .

Both DAGr (Gehrig and Scaramuzza, 2024) and EAGR process the spatio-temporal graphs G = { ν,E }, comprised of a set of nodes V connected by spatio-temporal edges E . Nodes in the graph include information about the spatial and temporal position of the event, which includes coordinates and time, and its feature given by polarity. Before being mapped into a node, an event’s spatial coordinates are normalized by the height and width, and the corresponding temporal feature $t_{i}$ is rescaled by a factor β . Each edge E in the graph links events that are close in both space and time, and the graph is directed to preserve the natural temporal order of events.

### 4.4 Augmentation

Data augmentation can increase the generalization ability of neural networks and greatly affect their performance (Zoph et al., 2020). The most common augmentation techniques for event-based data are similar to those used for traditional frame-based images and include horizontal flipping, zoom-in, zoom-out, resizing, adding noise, shearing, and cropping (Gehrig and Scaramuzza, 2023; Peng et al., 2023b).

On the other hand, other augmentation methods exploit the nature of event-based data for augmentation. EventDrop (Gu F. et al., 2021) is applied to raw events. It augments asynchronous event data by selectively removing events based on predefined strategies such as random drop, drop by time, and drop by area. The method was evaluated using DNN models with four event encoding representations, such as Event Frame, Event Count, Voxel Grid, and Event Spike Tensor (EST), on N-Caltech101 and N-Cars datasets. In addition, EventDrop can enhance the model’s generalization in object recognition and tracking by generating partially occluded cases, improving performance in scenarios with occlusion. Besides, EventDrop is reported to be compatible with SNNs too.

Similar to EventDrop, the EventMix method can be applied to both DNNs and SNNs. It creates augmentation by mixing event streams with a Gaussian Mixture Model (Shen G. et al., 2023). Performance of EventMix was tested on DVS-CIFAR10, N-Caltech101, N-CARS, and DVS-Gesture datasets. SNN with Event-Mix achieved state-of-the-art results (Shen G. et al., 2023).

Neuromorphic Data Augmentation (NDA), a family of geometric augmentations, was specifically designed to enhance the robustness of SNNs (Li Y. et al., 2022). SNN model with NDA improved accuracy by 10.1% and 13.7% on DVS-CIFAR10 and N-Caltech 101, respectively. The next ViewPoint Transform and Spatio-Temporal Stretching (VPT-STS) augmentation method is also designed for SNNs (Shen H. et al., 2023). In particular, the SNN model with VPT-STS achieved 84.4% on the DVS-CIFAR10 dataset. The VPT-STS generates samples from different viewpoints by transforming the rotation centers and angles in the spatiotemporal domain.

Another proposed method for enhancing event data diversity is Shadow Mosaic (Peng et al., 2023a). It consists of several stages, including Shadow Mosaic, Scaling, and Cropping, which aim to reduce the imbalance in spatio-temporal density of event streams due to different speeds of objects and the brightness change. Sparse shadow events are generated through random sampling, while dense shadow events are created by replicating events in the three-dimensional domain. At the mosaic stage, resulting shadow event samples are merged and scaled up or down, leading to a distortion. To restore realistic event structures, the shadow method is re-applied, and cropping is performed. The Shadow Mosaic augmentation method was used with Hyper Histograms encoding for the DNN model and improved mAP by up to 9.0% and 8.8% compared to the baseline without augmentation on the 1MP and GEN1 real-world datasets, respectively. A recent work introduced Random Polarity Suppression (RPS) augmentation method, which was applied on the VTEI tensor (Silva et al., 2025). Table 7 provides summary on augmentation techniques mentioned above.

**TABLE 7:** Augmentation techniques (* - after transforming events to frame-based format).

Augmentation	Frame-based	Event-based	Description
Flipping Gehrig and Scaramuzza (2023), Peng et al. (2023b)	✓	✓ *	Horizontal (left-right) or Vertical (Up-Down) mirroring of the image
Zooming Gehrig and Scaramuzza (2023), Peng et al. (2023b)	✓	✓ *	Rescaling and resizing image
Resizing Gehrig and Scaramuzza (2023), Peng et al. (2023b)	✓	✓ *	Resizing image
Cropping Gehrig and Scaramuzza (2023), Peng et al. (2023b)	✓	✓ *	Random cropping and extracting random sub-regions from images
Shearing Gehrig and Scaramuzza (2023), Peng et al. (2023b)	✓	✓ *	Slight distortions of images
Event-Drop Gu F et al. (2021)	✗	✓	Selectively removing events based on predefined strategies
Event-Mix Shen et al. (2023a)	✗	✓	Created by mixing event streams with Gaussian Mixture Model
NDA Li Y et al. (2022)	✗	✓	Geometric augmentations
VPT-STS Shen et al. (2023b)	✗	✓	Generates samples from different viewpoints
Shadow Mosaic Peng et al. (2023a)	✗	✓	Sparse and dense shadow events are generated and combined
RPS Silva et al. (2025)	✗	✓	Generated by randomly suppressing all events of a particular polarity

### 4.5 Hardware accelerators

#### 4.5.1 Graphical Processing Units

Majority of the event-based data object detection architectures with the state-of-the-art performance were trained and evaluated on Graphical Processing Units (GPUs), which represent conventional Von-Neumann architectures. Some of the works omit the hardware specification, making their direct comparisons challenging, but the most commonly used evaluation platforms for both dense and sparse algorithms include NVIDIA Tesla T4, NVIDIA Titan Xp, NVIDIA Quadro RTX 4000, and others (Gehrig and Scaramuzza, 2023; Peng et al., 2024). Generally, GPUs, along with specialized libraries such as PyTorch and TensorFlow, are well-suited for executing traditional DNNs due to their optimized support for parallel matrix operations and high computational throughput. However, they are less efficient when it comes to processing sparse models, as they typically do not skip computations involving zero-value elements (Smagulova et al., 2023).

Generally, sparse neuromorphic models like SNN are better aligned with the nature of event-based data, offering greater potential for efficient processing due to their ability to exploit data sparsity and reduce unnecessary computations. The same characteristic also poses a major obstacle to training efficiency. To address the issue, a range of specialized frameworks for SNNs have been developed, which include snnTorch and SpikingJelly, each targeting different aspects of model design and simulation. More recently, temporal fusion has been proposed as a strategy for scalable, GPU-accelerated SNN training Li et al. (2024).

#### 4.5.2 FPGA-based accelerators

AI-based object detection systems on FPGAs lag behind GPU-based developments due to a time-consuming implementation process(Kryjak, 2024). Additional challenges include the lack of standardized benchmarks and the limited availability of Hardware Description Language (HDL) codes. However, the introduction of Prophesee’s industry-first event-based vision sensors, combined with the FPGA-based AMD Kria Vision AI Starter Kit, marks a significant milestone for future advancements in the field (Kalapothas et al., 2022). The recent work introduces SPiking Low-power Event-based ArchiTecture (SPLEAT) neuromorphic accelerator, a full-stack neuromorphic solution that utilizes the Qualia framework for deploying state-of-the-art SNNs on an FPGA (Courtois et al., 2024). In particular, it was used to implement a small 32-ST-VGG model, which achieved 14.4 mAP on the GEN1 dataset. The model’s backbone was accelerated on SPLEAT, operating with a power consumption of just 0.7 W and a latency of 700 ms, while the SSD detection head was executed on a CPU.

#### 4.5.3 Neuromorphic platforms

Neuromorphic processing platforms for SNNs remain in their early stages of development, but represent a significant area of ongoing research (Bouvier et al., 2019; Smagulova et al., 2023). The notable SNN accelerators include Loihi (Davies et al., 2018), Loihi-2 (Orchard et al., 2021), TrueNorth (Akopyan et al., 2015), BrainScaleS (Schemmel et al., 2010), BrainScaleS-2 (Pehle et al., 2022), Spiking Neural Network Architecture (SpiNNaker) (Furber and Bogdan, 2020), SpiNNaker 2 (Huang et al., 2023), and one of the first commercially available neuromorphic processors, Akida by BrainChip (Posey, 2022).

TrueNorth is an early large-scale neuromorphic ASIC designed for SNNs. While it was a significant milestone in brain-inspired computing, it lacks the flexibility required for modern AI applications and has been superseded by newer designs. BrainScaleS and BrainScaleS-2 are mixed-signal brain-inspired platforms suitable for large-scale SNN simulations. However, their large physical footprint and complex infrastructure requirements make them less suitable for deployment in embedded or real-world applications such as autonomous driving Iaboni and Abichandani (2024).

CarSNN is a neuromorphic solution designed for classifying cars versus other objects using data from a ATIS sensor and an SNN deployed on Intel’s Loihi neuromorphic research chip. The solution was evaluated on the N-CARS dataset with an accuracy of 82.99%. The corresponding hardware implementation achieved a maximum latency of just 0.72 ms per sample while maintaining low power consumption at only 310 mW (Viale et al., 2021). Loihi supports on-chip learning and real-time SNN inference but offers limited scalability, whereas Loihi-2 is more suitable for real-world applications, including event-based object detection. Particularly, attention-based hybrid SNN-ANN backbone for event-based object detection achieved 0.35 mAP on the GEN1 dataset and 0.27 mAP on the 1Mp dataset (Ahmed et al., 2025). The same Hybrid SNN-ANN combined with RNN achieved 0.43mAP on GEN1. In this setup, the SNN component was accelerated on Loihi 2, delivering subreal-time performance while offering improved power efficiency compared to commercially available edge computing hardware (Ahmed et al., 2025). Temporally-binned Object Flow from Events (TOFFE) is an event-based object motion estimation framework. It achieved an 8.3 × reduction in energy consumption and a 5.8 × reduction in latency on a hybrid setup like Loihi-2 with Jetson TX2, compared to a 5.7 × energy and 4.6 × latency reduction on a standalone edge GPU (Jetson TX2), highlighting that Loihi-2 significantly contributes to improved efficiency and performance in event-based object detection. Kosta et al. (2025).

The demonstration of a fully neuromorphic solution based on the SpiNNaker platform equipped with ATIS camera was conducted for the visual tracking task (Glover et al., 2019). SpiNNaker and its successor SpiNNaker-2 are ARM-based processor platforms designed for simulating SNNs with a high degree of flexibility. However, their energy consumption is higher compared to dedicated circuit-based solutions like Loihi, making them less suitable for energy-constrained edge deployments (Yan et al., 2021).

As for the object detection task, a fully neuromorphic framework was deployed based on DVXplorer Lite camera by Inivation and Akida processor by Brainchip (Silva D. et al., 2024). This setup was specifically designed for edge computing, eliminating the need to transfer data to the cloud. Due to the constraints of the Akida chip, the YOLOv2 model was chosen and trained to detect cars, pedestrians, and two-wheelers from a synthetic dataset. Akida 2, the second generation of BrainChip’s neuromorphic processor, supports vision transformers, which made it even more suitable for event-based object detection and edge AI applications (BrainChip, 2025). Particularly, the recent demonstration of Akida 2 with Prophesee’s EVK4 event-based camera enables the integration of advanced visual intelligence into compact, low-SWaP (Size, Weight, and Power) devices Ltd (2025).

#### 4.5.4 Performance comparison

**FIGURE 9:**
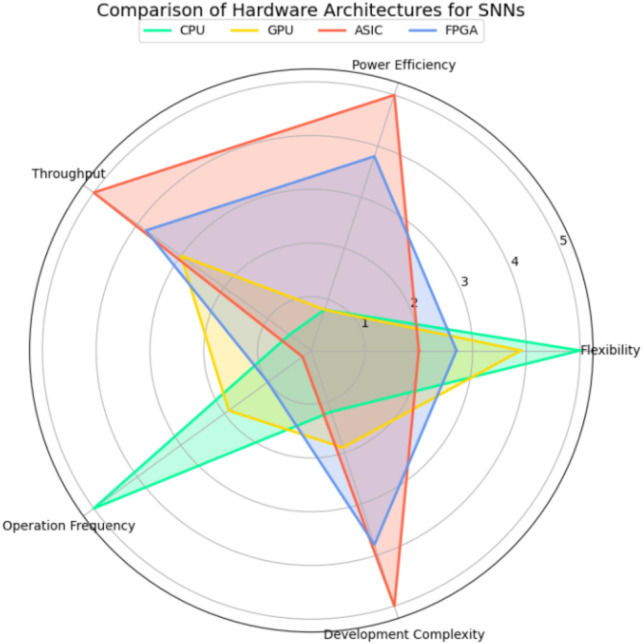
Performance comparison of hardware platforms for SNN acceleration (Isik, 2023).

There is a growing shift toward neuromorphic vision, driven by event-based sensors. Their output naturally aligns with neural-inspired SNNs. The performance differences among hardware platforms are emphasized in comparative studies of SNN acceleration across GPUs, Central Processing Units (CPUs), Field Programmable Gate Arrays (FPGAs), and Application-Specific Integrated Circuits (ASICs), which assess factors such as power efficiency, flexibility, development complexity, operating frequency, and throughput (Isik, 2023). The study results, illustrated in Figure 9, indicate that FPGA and ASIC platforms are particularly promising for accelerating SNNs in terms of power efficiency and throughput. However, their utilization remains challenging due to factors such as design complexity, limited programmability, and the need for specialized development tools.

SNN acceleration on neuromorphic hardware platforms promises ultra-low latency and energy efficiency, particularly making them attractive for real-time perception in autonomous driving Ltd (2025). Table 8 summarizes the performance comparison of different platforms in the implementation of object detection using YOLOv2 model Putra et al. (2025), which proves that spike-driven computation by Akida achieved the highest power/energy efficiency without consideration of accuracy.

**TABLE 8:** Object detection using YOLOv2 on various platforms Putra et al. (2025).

Platform	Performance, FPS	Power, W	Efficiency, FPS/W
Desktop CPU: Intel i7-6700HQ	78.2	29.88	2.62
Desktop GPU: Nvidia GTX 960 M	219.7	46.67	4.71
Embedded CPU: ARM Cortex-A57	0.23	4.00	0.06
Embedded GPU: Nvidia Jetson TX2	7.8	1.02	40.81
FPGA: ZedBoard ZCU102	5.8	1.20	4.50
FPGA: Virtex-7 XC7V690t	302.3	11.35	26.63
Akida Neuromorphic Platform	6.0	0.078	76.92

However, the widespread adoption of neuromorphic platforms in the near future is limited by an immature ecosystem, the absence of standardized software toolchains, and a lack of comprehensive benchmarking against established GPU and FPGA platforms. Moreover, most of these platforms are not yet commercially available and remain primarily confined to research settings Putra et al. (2025).

## 5 System-level evaluation of event-based detectors

### 5.1 Performance of the state-of-the-art models

Being among the first real-world large-scale datasets for event-based vision, GEN1 and 1MP have achieved widespread adoption and have established themselves as the primary benchmarks for detection models evaluation.

The recently introduced eTRaM dataset addresses its limitations by providing higher-quality event data, more complex traffic scenarios, and includes annotations for detection, tracking, and motion prediction tasks. Table 9 provides the summary on the state-of-the-art event-based object detectors and their performance on these datasets, respectively. The table contains only reported results, thereby highlighting the lack of standardization and complicating fair comparisons. The primary goal of these architectures for event-based object detection is to develop lightweight models that can efficiently process spatio-temporal data.

**TABLE 9:** State-of-the-art event-based object detectors and their performance.

Type of the model	Object detector	Modality	Backbone	Head	Embedding	Augmentation	Params (M)	GEN1			1 Megapixel			eTraM		
								mAP50:95	Runtime	FLOPS (G)	mAP50:95	Runtime	FLOPS (G)	mAP50:95	Runtime	FLOPS (G)
Asynchronous	MatrixLSTM Cannici et al. (2020)	Events	RNN + CNN	YOLOv3	Raw Events	n/a	61.5	31	n/a	n/a	n/a	n/a	n/a	n/a	n/a	n/a
	AsyNet Messikommer et al. (2020)	Events	Sparse CNN	YOLOv1	2D Hist	n/a	11.4	14.5	n/a	n/a	n/a	n/a	n/a	n/a	n/a	n/a
	AEGNN Schaefer et al. (2022)	Events	GNN	YOLOv1	Raw Events	n/a	20.0	16.3	n/a	n/a	n/a	n/a	n/a	n/a	n/a	n/a
	ASTMNet Li J et al. (2022)	Events	(T)CNN + RNN	SSD	Raw Events	n/a	>100	46.7	35.6	n/a	48.3	72.3	n/a	n/a	n/a	n/a
	Spiking-YOLO Kim et al. (2020)	Events	SNN	YOLOv3	HIST	n/a	n/a	- (44.22 mAP50)	n/a	n/a	n/a	n/a	n/a	n/a	n/a	n/a
	Tr-Spiking-YOLO Yuan et al. (2024)	Events	SNN	YOLOv3	Voxel Grids with discretized time domain	n/a	8.7	- (45.34 mAP50)	n/a	n/a	n/a	n/a	n/a	n/a	n/a	n/a
	EAS-SNN (M) Yuan et al. (2024)	Events	SNN	YOLOX	ARSNN	random zoom-in, horizontal flipping	25.3	40.9 (73.1 mAP50)	n/a	n/a	36.2 (65.1 mAP50)	n/a	n/a	n/a	n/a	n/a
	EAGR Gehrig and Scaramuzza (2024)	Events	CNN + GNN	YOLOX	G = { ν,E }	augmented graph	n/a	14.1	n/a	n/a	n/a	n/a	n/a	n/a	n/a	n/a
	DAGR Gehrig and Scaramuzza (2024)	Events + Frames	ResNet-50+GNN	YOLOX	G = { ν,E }	augmented graph random horizontal flipping, random magnification	n/a	41.9	n/a	n/a	n/a	n/a	n/a	n/a	n/a	n/a
	MHSANet-YOLO Fan et al. (2025)	Events	MHSANet	YOLOX	Raw Events	n/a	76.2	76.7	n/a	n/a	n/a	n/a	n/a	n/a	n/a	n/a
	RED Perot et al. (2020)	Events	CNN + RNN	SSD	Event Volume (50 ms)	n/a	24.1	40	16.7 (Titan Xp)	6.0	43.0	39.3 (Titan Xp)	19.0	n/a	n/a	n/a
	AED (Liu et al., 2023)	Events	adapted Darknet21	YOLOX	TAF	random flipping random cropping random resizing	14.8	45.4	11.98 (Titan Xp)	n/a	34.4	13.36 (Titan Xp)	n/a	n/a	n/a	n/a
Fixed rate	RVT-B Gehrig and Scaramuzza (2023)	Events	Transformer (MaxViT)+ RNN	YOLOX	SHIST (50 ms)	random horizontal flipping, zooming-in, zooming-out	18.5	47.2	10.2 (T4 GPU)	3.5	47.4	11.9 (T4 GPU), 16.0 (Titan Xp)	10.3	29.5	11.9	10.3
	SAST Peng et al. (2024)	Events	Transformer (MaxViT)+ RNN	YOLOX	SHIST (50 ms)	random horizontal flipping, zooming-in, zooming-out	18.9	47.9		2.1	48.3	19.7 (Titan Xp)	5.6	30	24.4	6.2
	SSM Zubic et al. (2024)	Events	Transformer + (S4, S5) SSM	YOLOX	SHIST (50 ms)	random horizontal flipping, zooming-in, zooming-out	18.2	47.7	8.16 (T4 GPU)	n/a	47.8	9.57 (T4 GPU)	n/a	29.3	10.9	>9.1
	Swin-T v2 (Liu et al., 2022)	Events	Transformer + RNN	YOLOX	Patched Voxel Grid	n/a	21.1	45.5	26.6	n/a	46.4	34.5	n/a	n/a	n/a	n/a
	Nested-T (Peng et al., 2023b)	Events	Transformer + RNN	YOLOX	Patched Voxel Grid	n/a	22.2	46.3	25.9	n/a	46	33.5	n/a	n/a	n/a	n/a
	GET Peng et al. (2023b)	Events	Transformer + RNN	YOLOX	Group Token (50 ms)	n/a	21.9	47.9	16.8 (GTX 1080Ti)	n/a	48.4	18.2 (GTX 1080Ti)	n/a	n/a	n/a	n/a
	ERGO-12 (Zubić et al., 2023)	Events	Transformer	YOLOv6	ERGO-12	Mixup and Mosaic	n/a	50.4	n/a	n/a	40.6	n/a	n/a	n/a	n/a	n/a
	ReYOLOv8(m) (Silva et al., 2025)	Events	CNN + RNN	YOLOv8	VTEI	flipping, zooming-in, zooming-out, RPS	18.1	49.4	15.5 (v100)	n/a	n/a	n/a	n/a	n/a	n/a	n/a
	SMamba (Yang et al., 2025)	Events	SSM + RNN	YOLOX	SHIST (50 ms)	random horizontal flipping, zooming-in, zooming-out	16.1 (GEN1) 16.7 (1MP, eTraM)	50.4	24.0	2.4	49.3	26.0	7.4	32.6	25.2	6.6
Other	Hybrid SNN-ANN (Ahmed et al., 2024)	Events	hybrid + RNN	YOLOX	Raw Events	resize, crop, flip	7.7	43.0	n/a	n/a	n/a	n/a	n/a	n/a	n/a	n/a
	HTMNet (Hamaguchi et al., 2023)	Events	HTMNet-B1	YOLOX-Lite	ESCA	resize, crop, flip	n/a	45.5	4.6	n/a	n/a	n/a	n/a	n/a	n/a	n/a
		Events	HTMNet-L1	YOLOX-Lite	ESCA	resize, crop, flip	n/a	47.0	5.6	n/a	n/a	n/a	n/a	n/a	n/a	n/a
		Events	HTMNet-B3	YOLOX-Lite	ESCA	resize, crop, flip	n/a	45.2	7.0	n/a	n/a	n/a	n/a	n/a	n/a	n/a
		Events	HTMNet-L3	YOLOX-Lite	ESCA	resize, crop, flip	n/a	47.1	7.9	n/a	n/a	n/a	n/a	n/a	n/a	n/a
	ChimeraNet-3M (Ahmed et al., 2024)	Events	mixed blocks	YOLOv8	SHIST	resize, crop, flip	3	44.6	n/a	n/a	n/a	n/a	n/a	n/a	n/a	n/a
	ChimeraNet-5M (Ahmed et al., 2024)	Events	mixed blocks	YOLOv8	SHIST	resize, crop, flip	5	46.0	n/a	n/a	n/a	n/a	n/a	n/a	n/a	n/a
	ChimeraNet-10 M (Ahmed et al., 2024)	Events	mixed blocks	YOLOv8	SHIST	resize, crop, flip	10	47.7	n/a	n/a	n/a	n/a	n/a	n/a	n/a	n/a

### 5.2 End-to-end evaluation

Most event-based algorithms process a fixed number of events at each step, typically using a fixed time window $t_{w}$ . When raw event data needs to be converted into an intermediate representation, typically to be processed by dense and graph-based models, this step can significantly affect performance by introducing distortions and delays. These effects can be measured using parameters such as the time-windows $t_{w}$ , conversion time $t_{ec}$ , and data compression rate. During the inference stage, the conversion time $t_{ec}$ is typical for dense and graph-based models, but is absent in asynchronous and SNN models that process raw events directly.

In (Gehrig et al., 2019), the authors demonstrated that the representation computation time $t_{ec}$ contributed only a small fraction to the overall processing time, which was dominated by model inference. Specifically, for a 100 ms sample from the N-Cars dataset, the representation step took just 0.38 ms, whereas the total computation time ranged from 4.25 ms to 6.08 ms, depending on the model’s complexity. Notably, the representation was computed on the CPU, while inference ran on the GPU. Nevertheless, most of the other works on event-based object detection did not report the time required for computing the event representation. This omission is critical because representation computation can introduce non-negligible latency in real-time applications and more complex data.

The impact of the size of $t_{w}$ , the duration over which these events are aggregated for processing, and also known as “integration time”, was studied in (Silva et al., 2024c; Maqueda et al., 2018). During the evaluation of the GEN1 dataset using the YOLOv5 model with attention, it was observed that varying $t_{w}$ between 10-125 ms had an impact on performance (Silva et al., 2024c). Specifically, smaller $t_{w}$ values were more effective for detecting low-speed, smaller objects such as pedestrians, while increasing $t_{w}$ improved detection of higher-speed objects like cars. Similarly, (Maqueda et al., 2018), evaluated five integration times and identified 50 ms as the optimal value. Additionally, $t_{w}$ also impacts noise accumulation (Silva D. et al., 2024). Besides, the volume of encoded data and the memory size required for storage and processing are not typically reported.

After converting the raw event data into a specific format suitable for processing, the model generates a set of preliminary predictions based on this input. These predictions typically include multiple overlapping bounding boxes for detected objects. To refine the results and eliminate redundant detections, a Non-Maximum Suppression (NMS) post-processing step is applied. NMS works by selecting the bounding box with the highest confidence score and suppressing all other boxes with significant overlap (as measured by Intersection over Union, IoU). This ensures that each detected object is represented by a single, most accurate bounding box.

Overall, a neuromorphic object detection system requires full integration of the entire processing pipeline, including event stream preprocessing, model training, and the final detection stage. The training pipeline time can be represented as in Equation 16 below:

$$t=t_{w}+t_{ec}+t_{\mathrm{train}}+t_{mc}$$ (16)

where $t_{w}$ is the intagration time-window, $t_{ec}$ is the time required for converting the events to an intermediate format, $t_{\mathrm{train}}$ is the training time of the model in GPU hours, and $t_{mc}$ is an optional stage and shows time required for model format conversion.

Similarly, as in Equation 17 the total computation time during inference can be summarized by:

$$t=t_{w}+t_{ec}+t_{\mathrm{eval}}+t_{\mathrm{nms}}$$ (17)

where $t_{w}$ is the integration time window, $t_{ec}$ is the time required for converting the events to an intermediate format and optional for certain models, $t_{\mathrm{eval}}$ is the processing time throughout the model, and $t_{\mathrm{nms}}$ is the duration of the NMS post-processing.

As can be seen from Table 9, most of the works report only performance parameters during the processing of the model, excluding the processing step of adapting events to the required representation format, like frame or graph. In this work, the evaluation of the system throughput is included as part of the survey and summarized in Table 10. The results were obtained using 100 randomly chosen samples from the GEN1 and 1MP datasets on an RTX 4090 24GB GPU. Particularly, each sample consists of 60 s recordings Perot et al. (2020). Given 50-ms time windows in video slices, 100 samples result in 120,000 image samples. The tests were performed with a warm-up phase of 30 epochs. We used a batch size of eight, which is the most common size used in literature, and multiplied the batch throughput by eight to obtain the image throughput. However, SSMS (Base) model encountered out-of-memory (OOM) issues. Alternatively, the SSMS (Small) variant was used instead. Similarly to RVT (Figure 7a), both SSMS (Figure 7c) is based on the transformer architecture and additionally employs the same SHIST encoding. However, RVT did not suffer from OOM. In the RVT, spatial and temporal feature aggregation are handled separately, with vanilla LSTM layers placed at the end of each block to model temporal dependencies. The use of LSTM cells slows down training, and the resulting weights tend to generalize only to data sampled at the same frequency as during training. On the other hand, SSMS offers adaptability to varying frequencies during inference without the need for retraining. SSMS utilizes S4 or S5 layers for temporal aggregation instead of LSTM. SSMs enable parallel, efficient long-sequence modeling by reducing compute bottlenecks through learned state-space kernels Somvanshi et al. (2025). As a result, the burden falls on GPU memory, and SSMS encounters OOM issues due to its long convolution kernels that generate large intermediate buffers, particularly with high-resolution images. LSTMs avoid this problem since they only keep a hidden state at each step.

**TABLE 10:** System throughput of models from raw events to model prediction for 100 samples of GEN1 and 1MP datasets.

Model	Format	$t_{w}$	GEN1				1 Megapixel			
			Throughput (FPS)	Params (M)	MACs (G)	FLOPs (G)	Throughput (FPS)	Params (M)	MACs (G)	FLOPs (G)
RVT (Gehrig and Scaramuzza, 2023)	SHIST	50	46.00	18.50	5.1	65.6	10.2	18.5	15.2	30.5
SAST (Peng et al., 2024)	SHIST	50	24.72	18.81	1.5	3.1	53.92	18.81	4.7	9.4
SSMS (Small) (Zubic et al., 2024)	SHIST	50	33.04	18.19	97.5	200.0	-	18.19	1100	2300
SMamba (Yang et al., 2025)	tokens	n/a	19.76	16.07	4.7	9.5	34.8	16.67	14.3	28.7
EAS-SNN (Wang H et al., 2024)	raw events	n/a	21.36	25.28	86.2	172.8	21.36	25.28	86.2	172.8
Obj Det. SNN ()	raw events	n/a	24.16	12.65	12.8	25.7	-	-	-	-
SODF (Li et al., 2023)	event image	n/a	22.68	8.2	13.4	26.8	-	-	-	-
GET (Peng et al., 2023b)	Group Token	n/a	31.48	4.58	1.56	3.2	-	-	-	-

Overall, it can be seen from Tables 9, 10 that the required number of FLOPs and MACs increased when event encoding was included. For example, processing RVT without encoding required 3.5 GFLOPs, whereas with encoding, it increased to 10.2 GFLOPs. In the case of SAST, the increase was lower from 2.5 GFLOPs without encoding to 3.5 GFLOPs with it. This again highlights the importance of carefully considering event encoding, as it can significantly affect not only performance but also the computational cost, depending on the model.

## 6 Discussion and future directions

In the realm of event-based vision, autonomous driving is one of the most prominent applications as it demands high-speed motion handling, low-latency perception, and reliable operation under challenging lighting conditions (Chen et al., 2020). This work surveys an end-to-end pipeline for the implementation of event-based object detection, starting from types of event-based sensors to the performance of the state-of-the-art models.

### 6.1 Datasets

As reflected in the survey results, event-based data remains underrepresented in data science and machine learning research, with a notable absence of standardized benchmarks for evaluating encoding techniques and model performance. Initially, DVS-converted datasets were used to compensate for the lack of event-based data. But these datasets generally exhibit lower sparsity and more uniform distributions compared to DVS-captured data, which more accurately represent real-world scenes. Development of synthetic datasets can be useful for pre-training models, which can then be fine-tuned on real-world data for improved performance.

In addition, current event-based datasets lack a diverse range of object classes necessary to support full automation in Autonomous Driving Systems. Future work should prioritize the collection of more comprehensive data, including a broader set of classes relevant to real-world driving scenarios, including on-road and off-road. This includes dynamic agents such as pedestrians, cyclists, motorcyclists, cars, vans, buses, and trucks, as well as traffic infrastructure like signs, lane markings, crosswalks, and others. Additionally, the system must recognize temporary or rare obstacles such as construction equipment, road debris, and emergency vehicles. Contextual awareness of sidewalks, curbs, vegetation, and buildings further enhances scene understanding.

### 6.2 Sensors fusion

The collection of high-quality real-world event-based datasets requires advancements in current event camera technology, particularly in terms of control capabilities. Existing bias settings in event-based cameras are often insufficient to effectively manage noise, limiting data quality in complex environments. One of the key future directions should be the improvement of the controllability of event cameras.

Additionally, event-based vision systems face challenges in detecting static objects due to their motion-dependent sensing, highlighting the need for improvement and ensuring robust perception. One approach to overcoming this limitation is through sensor fusion of Dynamic Vision Sensors (DVS) and Active Pixel Sensors (APS), as demonstrated in DAVIS cameras Shawkat et al. (2024) or putting DVS and frame-based cameras side by side Perot et al. (2020). In addition, a setup that integrates event-based sensors with complementary sensing modalities such as LiDAR, radar, and inertial measurement units (IMU) can further enhance perception capabilities Gehrig et al. (2021), Zhu et al. (2018a), Chaney et al. (2023). The next is a multi-view setup, where two or more event cameras capture a static object from different viewpoints, as in the DSEC dataset Gehrig et al. (2021). Particularly, in multi-modal datasets that include MVSEC Zhu et al. (2018a), DSEC Gehrig et al. (2021), and M3ED Chaney et al. (2023), static objects are mostly captured through ego-motion or sensor fusion. Similarly, SEVD represents a multi-view synthetic vision-based cooperative setup, where ego and fixed perception are combined Aliminati et al. (2024). FlexNet is a framework that integrates high-frequency event data with semantic information from RGB frames to enable object detection in both fast-moving and static scenarios Lu et al. (2024). Nevertheless, its performance gains over state-of-the-art methods are limited to the frequency range of 20–180 Hz.

Challenges in sensor fusion arise from spatial calibration and temporal synchronization, since event-based sensors produce asynchronous outputs, whereas frame-based cameras, LiDAR, radar, and IMUs typically operate at synchronous, fixed rates. Moreover, these modalities differ in output format and resolution, complicating fusion. Finally, deploying multiple sensing architectures increases both power consumption and hardware footprint. Therefore, while fusing event-based cameras with complementary modalities such as IMU, LiDAR, and radar, RGB can help overcome the challenge of detecting static objects, it also introduces cost, calibration requirements, and system complexity Gehrig et al. (2021), Lu et al. (2024). As an alternative approach to static object detection, compensation algorithms can be introduced, for example, by generating pseudo-labels for non-moving objects Messikommer et al. (2022).

### 6.3 Models

Recent progress in event-based vision underscores the unique benefits of asynchronous sensing; however, existing object detection models still underexploit the potential of event data. This gap stems largely from the reliance on frame-centric design principles, which do not align naturally with the sparse and continuous characteristics of event streams.

Currently, only a limited number of architectures are capable of natively handling event-based inputs. Spiking Neural Networks (SNNs) and Graph Neural Networks (GNNs) have emerged as promising candidates due to their ability to process asynchronous signals and non-Euclidean structures, respectively. Nevertheless, evaluations of these approaches remain confined to relatively simple benchmarks, such as GEN1, while their applicability to more demanding large-scale datasets (e.g., 1MP and eTraM) has not yet been demonstrated.

SNNs, in particular, face challenges in direct training due to the non-differentiability of spike generation functions. To mitigate this, several pipelines rely on training conventional deep neural networks followed by conversion into spiking counterparts, a process that introduces additional complexity and often compromises efficiency. GNN-based approaches, on the other hand, depend on transforming events into graph structures; however, this representation does not naturally capture the continuous temporal dynamics of event streams, leading to suboptimal performance. As a result, the most competitive results in event-based detection are still achieved using dense models that reformat events into frame-like structures, subsequently processed with CNNs or Transformers. While effective, these strategies diminish the temporal fidelity and sparsity advantages inherent to event cameras.

Addressing these limitations requires improving model scalability and developing systematic methods to identify architectures that are inherently well-suited to event-driven data. Recent advances in scalable training mechanisms and automated architecture search present promising directions in this regard.

#### 6.3.1 Scalability

Scalability constitutes a central bottleneck in extending event-based models to real-world applications. In the case of SNNs, surrogate gradient methods have been instrumental in enabling stable backpropagation through spiking activity, thereby supporting deeper and more expressive architectures Su et al. (2023), Fan et al. (2025). These algorithmic advances, when paired with emerging neuromorphic hardware platforms such as Intel Loihi 2 and SpiNNaker 2, provide new opportunities for efficient large-scale training and inference.

For GNNs, the computational cost of message passing across large, dynamic event graphs remains prohibitive. Sampling-based strategies provide a path forward: cluster-based sampling facilitates hardware-friendly partitioning for efficient event-to-graph conversion and real-time inference Chiang et al. (2019), while neighborhood sampling reduces training overhead by restricting aggregation to local regions of interest Yang et al. (2024). Additionally, stochastic subgraph sampling methods, such as GraphSAINT, improve scalability by lowering variance and complexity without sacrificing representational power Zeng et al. (2019).

More recently, hybrid approaches integrating sampling with spatiotemporal attention mechanisms have demonstrated improved scalability for event-driven GNNs, highlighting the potential of combining structural sparsity with adaptive temporal modeling. These efforts collectively emphasize that scalability solutions must be tailored to the asynchronous and sparse nature of event-based signals rather than directly borrowing from frame-based paradigms.

#### 6.3.2 NAS

Neural Architecture Search (NAS) offers a principled framework for automatically identifying architectures optimized for event-driven data. Unlike hand-crafted models, NAS can efficiently explore large design spaces, balancing task-specific accuracy with computational efficiency. Within event-based vision, early frameworks such as Chimera-NAS have shown the feasibility of tailoring architectures to asynchronous modalities Silva et al. (2024b).

Looking forward, extending NAS methodologies to support SNNs and GNNs represents an important research direction. Such extensions would allow the automatic discovery of models that are not only well-suited to the temporal sparsity of event data but also optimized for emerging neuromorphic hardware. Hybrid pipelines combining CNN, SNN, and GNN components could also be jointly optimized through NAS to achieve improved trade-offs across accuracy, latency, and energy efficiency. Furthermore, hardware-in-the-loop NAS, where the search process directly incorporates constraints from neuromorphic accelerators, has the potential to further align architectural design with deployment feasibility.

### 6.4 Hardware

The strong performance of dense models is largely enabled by the high computational power and parallel processing capabilities of GPUs. On the other hand, the research in neuromorphic hardware is rapidly growing, driven by its demonstrated advantages in reducing latency and enhancing power efficiency. Nevertheless, it is crucial to continue improving the performance of asynchronous models that can process raw event data directly, as these models are particularly well aligned with the inherent characteristics of event data.

Solutions like SPLEAT and TOFFE also reflect the current trend toward hybrid hardware architectures that combine conventional CPU/GPU processing with neuromorphic platforms (Kosta et al., 2025; Courtois et al., 2024). Additionally, there is significant potential for developing hardware-aware NAS strategies that optimize architectures based on the constraints and capabilities of event-driven hardware platforms.

### 6.5 Encoding

Determining the most effective encoding format for event streams remains an unresolved challenge. Current practice shows that metrics such as throughput, memory usage, and the statistical distribution of encoded data are essential for meaningful comparisons Guo et al. (2021). Yet, most pipelines still rely on converting events into frame-like formats for compatibility with dense CNN or Transformer backbones. This approach is simple but introduces latency, discards fine temporal relationships, and can lead to information loss depending on the chosen frame rate.

A variety of alternative encodings have been proposed. Early works used grayscale reconstructions from event streams, while more recent approaches introduce time surfaces, voxel grids, or recurrent encoders such as ConvLSTMs Perot et al. (2020). Others, like the Agile Event Detector, adapt the encoding to motion speed, mitigating the limitations of fixed time windows Liu et al. (2023). Graph-based methods, such as AEGNN, preserve spatiotemporal continuity by incrementally updating event graphs rather than re-encoding entire frames.

Despite these advances, no single encoding strategy consistently outperforms others across datasets and tasks. Each representation trades off temporal fidelity, latency, and compatibility with downstream architectures. As noted in recent surveys, a systematic, large-scale evaluation of encoding methods under controlled conditions is still missing. Extensive simulations across diverse scenarios will therefore be essential to establish clear best practices.

### 6.6 Data augmentation

The training methodology of object detectors also impacts the final performance. Several studies suggest that incorporating data augmentation techniques can improve the accuracy of the models. Most data augmentation techniques used in event-based vision have been adapted from conventional frame-based processing and are typically applied after converting event data into frame-like representations. However, there are also augmentation methods specifically designed for event-based data, which can further improve performance in various vision tasks (Li Y. et al., 2022; Zoph et al., 2020). Further studies on augmentation techniques are required to improve model performance and adversarial robustness.

### 6.7 Evaluation and benchmarking

In addition to the lack of well-established models and accelerators, there is a gap in their fair evaluation. Specifically, reported results often fail to account for the throughput and memory requirements of encoding techniques for dense models. The runtime $t_{\mathrm{eval}}$ is influenced by factors such as model complexity, encoding format, and the GPU used. However, only a limited number of studies provided details about the GPU models used to train the models. This lack of transparency can lead to misleading conclusions about model performance. Besides, GPUs are designed for vector-based computations, which is useful in dense DNN models with large parameter count and Multiply-and-Accumulate (MAC) operations. However, neuromorphic hardware may better leverage the sparsity of event-based inputs and offer reduced computational cost, power consumption, and latency (Ahmed et al., 2025).

While this paper focused on enabling autonomous driving through the lens of object detection, achieving full vehicle autonomy, as defined by the six levels of automation, requires addressing a broader range of perception and decision-making tasks. Object detection is a foundational component, but additional capabilities such as semantic segmentation, instance segmentation, depth estimation, tracking, and scene understanding are essential for comprehensive environment modeling. These tasks enable more precise localization, obstacle avoidance, and dynamic path planning. Future work should therefore extend beyond object detection to develop and integrate these complementary functions, particularly in the context of event-based sensing, to move closer to robust, fully autonomous driving systems. Full autonomy will also require effective sensor fusion, combining event cameras with traditional RGB sensors, LiDAR, radar, and GPS to leverage the strengths of each modality. Besides, there is a need to study the robustness of these systems against adversarial attacks.

Finally, establishing standardized evaluation benchmarks and simulation tools for event-driven driving tasks will be crucial to accelerate research and ensure safe, real-world deployment. This can be promoted through the release of large-scale, open-access data under diverse environmental conditions, including multimodal datasets. The development of simulation platforms, such as CARLA with realistic event camera models, would further enable reproducible testing and facilitate comparison of algorithms. Additionally, there is a need to adopt unified evaluation protocols that include not only mAP and runtime, but also event throughput, energy per inference, and robustness under adverse conditions. Together, these efforts will promote consistency, reproducibility, and trustworthiness in evaluating event-based detection systems for autonomous driving.
